# The Central Complex as a Potential Substrate for Vector Based Navigation

**DOI:** 10.3389/fpsyg.2019.00690

**Published:** 2019-04-05

**Authors:** Florent Le Moël, Thomas Stone, Mathieu Lihoreau, Antoine Wystrach, Barbara Webb

**Affiliations:** ^1^Research Centre on Animal Cognition, Centre for Integrative Biology, CNRS, University of Toulouse, Toulouse, France; ^2^School of Informatics, University of Edinburgh, Edinburgh, United Kingdom

**Keywords:** vector, path integration, memory, insect, navigation, neural modeling, traplining, central complex

## Abstract

Insects use path integration (PI) to maintain a home vector, but can also store and recall vector-memories that take them from home to a food location, and even allow them to take novel shortcuts between food locations. The neural circuit of the Central Complex (a brain area that receives compass and optic flow information) forms a plausible substrate for these behaviors. A recent model, grounded in neurophysiological and neuroanatomical data, can account for PI during outbound exploratory routes and the control of steering to return home. Here, we show that minor, hypothetical but neurally plausible, extensions of this model can additionally explain how insects could store and recall PI vectors to follow food-ward paths, take shortcuts, search at the feeder and re-calibrate their vector-memories with experience. In addition, a simple assumption about how one of multiple vector-memories might be chosen at any point in time can produce the development and maintenance of efficient routes between multiple locations, as observed in bees. The central complex circuitry is therefore well-suited to allow for a rich vector-based navigational repertoire.

## 1. Introduction

It is well established that central place foraging insects, such as bees and ants, keep track of their displacement when they venture outside their nest by a process called path integration (PI) (Collett and Collett, 2000a,b). By combining compass and speed information, they continuously update a home vector that allows for a direct return to their nest after arbitrary outward routes (Müller and Wehner, 1988; Collett and Collett, 2000b). However, insects do not use their PI system only for homing. For instance, they can also store PI vector-memories and use them to return to a known food location (Wehner et al., 1983; Collett et al., 1999; Wolf and Wehner, 2000), and take shortcuts between multiple food locations (Menzel et al., 2005).

A recently published neural model (Stone et al., 2017) closely follows the connectivity of the insect Central Complex neuropil (CX) and uses properties of identified neurons in this circuit that respond to polarized light compass information and optic flow information to integrate an outbound path. In this model, the home vector, at any point in time, is assumed to exist as a distributed sinusoidal activity pattern across two sets of 8 columns, where the phase indicates direction, and amplitude indicates distance. The model also provides a mechanism for using such a PI memory to drive the animal directly back home. Offset connections between columns produce a comparison of the current heading to the home vector direction, and indicate whether steering left or right would improve the alignment. As the circuit continues to integrate movement, the home vector amplitude will decrease as it approaches the home position. When it becomes zero, an emergent search behavior will result, unless there is a mechanism to recognize home. The model accounts for changing travel speed and is also robust to decoupling between the agent body axis and direction of movement (Stone et al., 2017), something that bees (Riley et al., 1999), wasps (Stürzl et al., 2016) and ants (Pfeffer and Wittlinger, 2016; Collett et al., 2017; Schwarz et al., 2017) can do.

**Figure 1:**
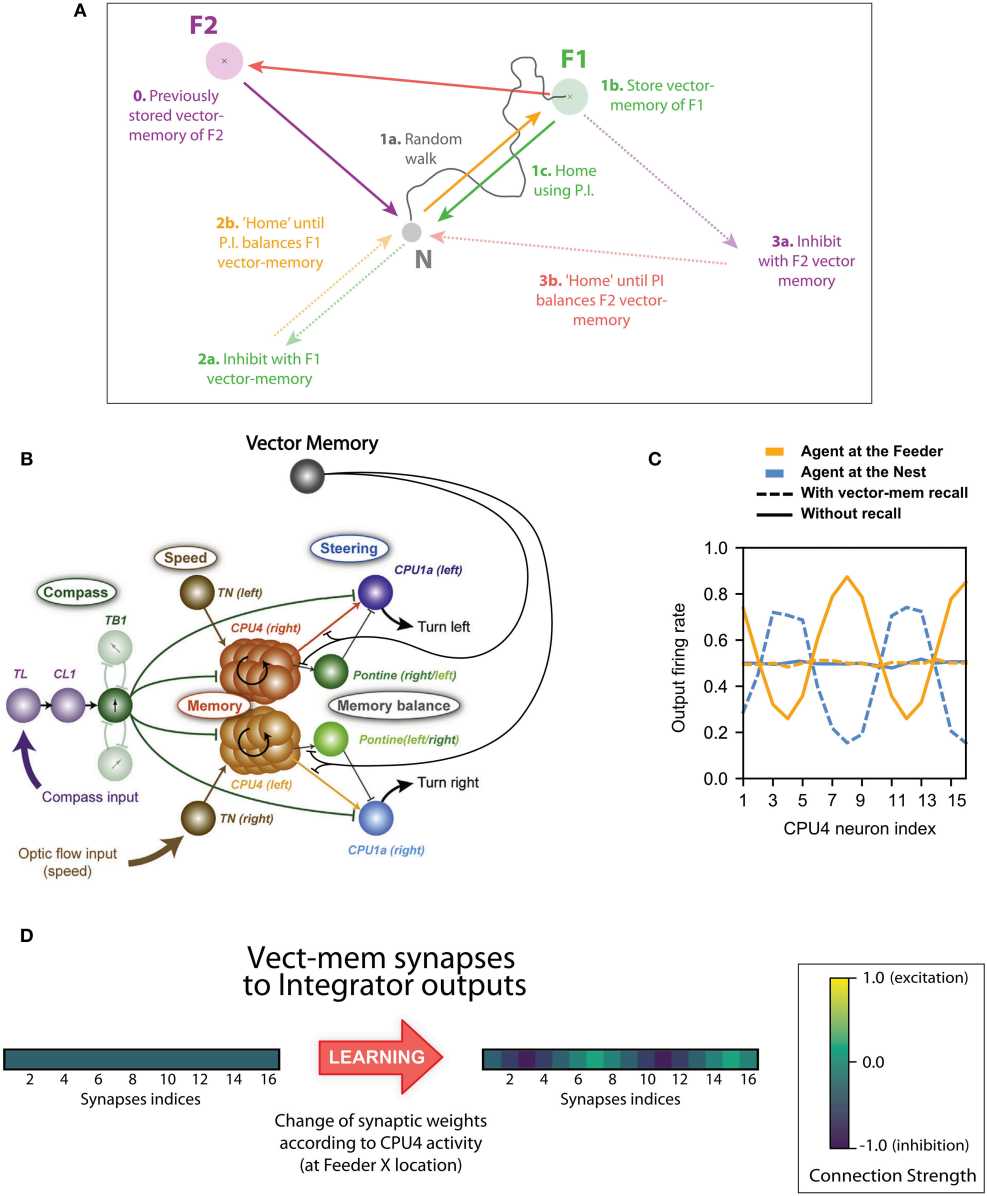
Basis of the concept of inhibition by Vector memory. **(A)** Example of the vector-memory and shortcut rationale: 0. The agent found a feeder (F2) on a previous trip and stored the corresponding home vector (solid purple) as a vector-memory. (1a) The agent leaves the Nest, performs a random walk (solid gray), and finds the feeder (F1). (1b) It stores the home vector (solid green) as a vector-memory. (1c) It uses the home vector to return to the Nest. (2a) The agent recalls the F1 vector-memory, “imagining” it is on the far side while actually at home (dashed green). (2b) It tries to “home” (dashed orange) which means it actually moves back to F1 (solid orange). (3a) At F1, no food is found: it lifts the recall of the F1 vector-memory and recalls the F2 vector-memory instead (dashed purple). (3b) It thus tries to “home” in a new direction (dashed red) which results in an actual movement from F1 to F2 (solid red). Lifting the F2 vector-memory recall allows it to home correctly (solid purple). **(B)** Principal connections of all cell types included in the Central Complex model: Shown are all connections of one direction cell (TB1), irrespective of columnar identity of individual cells (only two out of six connections to other TB1 cells are shown). The vector-memory neuron shows inhibitory synapses to the output fibers of the integrator (CPU4) cells, each of these synapses' weight being set according the corresponding CPU4 cell activity at the time of learning. **(C)** Example snapshots of the population activity of the 16 integrator (CPU4) neurons, at two different positions, with or without vector-memory recall: Solid lines thus correspond to the output of the integrator, dashed lines to the output of the integrator under the effect of a vector-memory neuron. At the Nest (solid blue), the integrator is in the zero-state (flat line). At the feeder (solid orange), the integrator encodes the position in polar coordinates across the population: sinusoid amplitude is the distance, phase is the angle. Under the inhibition by the vector-memory neuron, when the agent is at the Nest (dashed blue) the apparent coordinates encode for the Nest-to-Feeder vector. At the feeder, still under the effect of the vector-memory neuron (dashed orange), the integrator output and the inhibition cancel out, causing the apparent zero-state. **(D)** Example of the 16 synaptic weights of a vector-memory neuron, before and after learning: Before learning (leftmost vector), the synapses all have a weight of (negative) 0.5. After learning, some synapses get depressed toward 0 (inactive), others get reinforced toward negative 1.0. Each of these weights is changed according to the corresponding integrator (CPU4) cell activity at the time of learning.

The steering mechanism in this model is generalizable beyond the use of a home vector. Different sources of information about the “desired” heading or destination could be switched in, or additively combined onto the steering neurons, and the system will automatically steer to reduce the difference between the current and desired directions. While it is interesting to speculate how this might include information from sources other than PI (e.g., learnt terrestrial cues), here we focus on cases where the alternative activation is derived from a “vector-memory.” That is, we assume that, as in other models (Cruse and Wehner, 2011; Hoinville et al., 2012), the animal can store the current state of its home vector (the neural activation pattern) when it encounters salient places in its environment, and can later recover this vector-memory to guide future behavior (Figure 1A). We suggest some simple (hypothetical) neural circuitry that would add this capability to the CX model (Figure 1B) (we assess its biological plausibility in the discussion) and show it can support several interesting phenomena observed in insect navigation.

**Figure 2:**
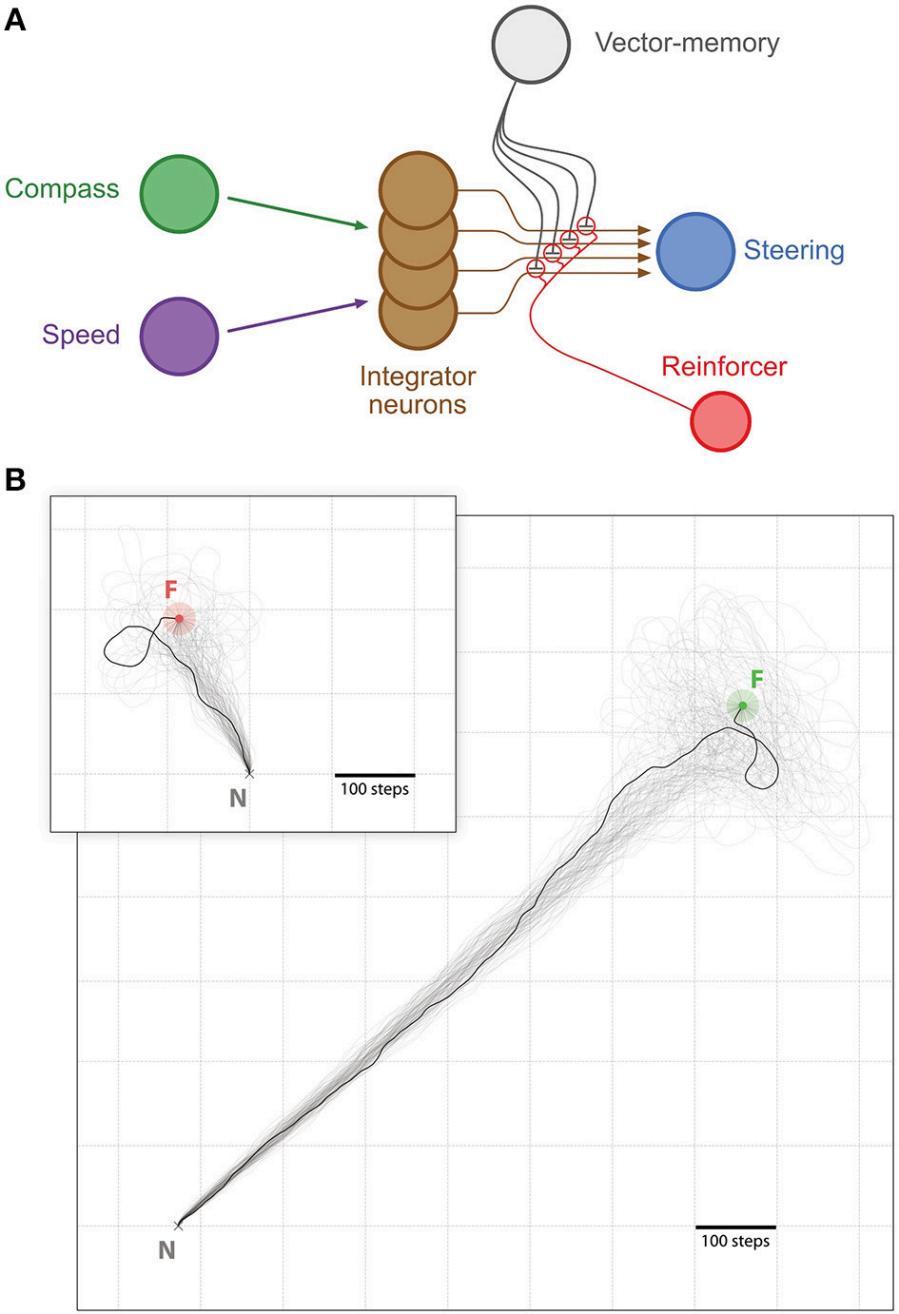
Memory-directed movement. **(A)** Simplified representation of the CX model with the vector-memory neuron. Layers before (Compass, green; Speed, purple) and after (Steering, blue) the integrator are represented as single nodes for simplicity. Only four integrator neurons (brown) are represented, with their output fibers. The vector-memory neuron (gray) synapses on each of these output fibers with inhibitory connections. These synapses' weights are set during learning according to the activity in the corresponding integrator output fiber, for example by a classic reinforcement process (Reinforcer neuron, red). **(B)** Examples of memory-directed movements: Large panel, distant Feeder (light green outer circle, Feeder catchment area; green inner circle, Feeder); Inset, Feeder close to the Nest (light red outer circle, Feeder catchment area; red inner circle, Feeder). In both examples, *n* = 100 individual paths (semi-transparent traces), with 1 more clearly marked. All paths are cut at 5,000 steps if the Feeder is not found.

**Figure 3:**
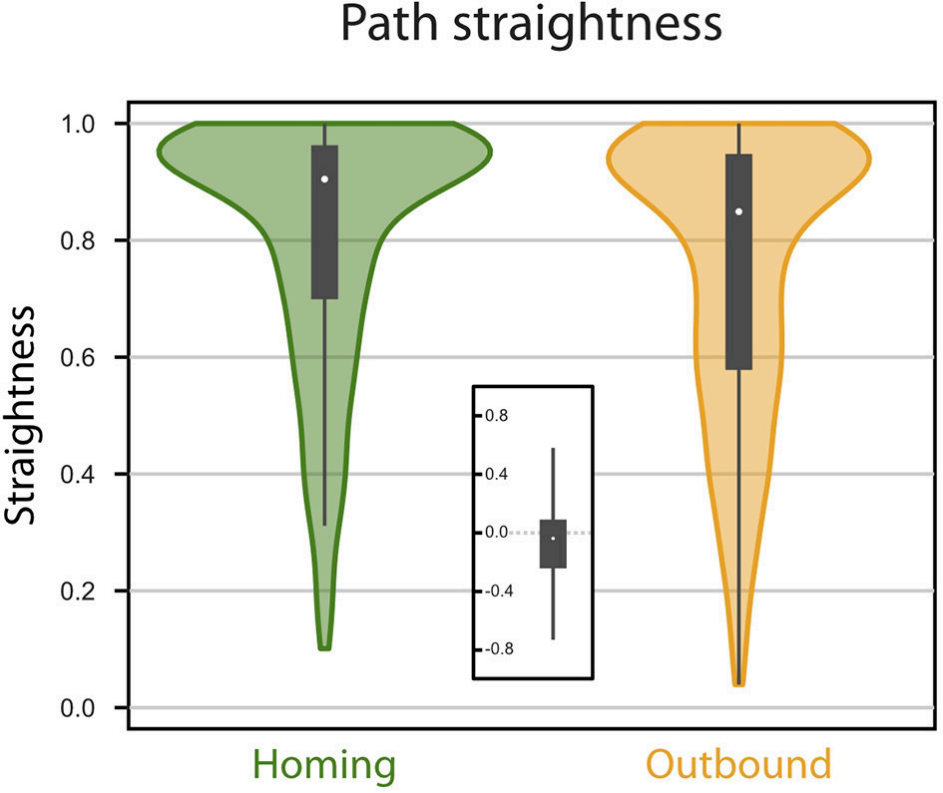
Path straightness. Violin Plots of the paths straightness. Straightness is given as the (bee-line) distance divided by the distance walked. Green, homing; orange, memory-directed foodward path. Thick gray bar, interquartile range; thin gray bar, 95% confidence interval; white dot, median. Inset indicates differences in path straightness (homing - foodward) for paired data (same random walk).

*Memory-directed movement*: Insects that have found a food source on a previous excursion can return to it on a direct route. It is assumed this involves storage of a memory of the PI state when the food was reached (Wehner et al., 1983; Collett et al., 1999; Wolf and Wehner, 2000). We hypothesize that such a memory could be integrated as a simple inhibitory influence in the CX steering circuit to produce food-ward steering and search around the food location (Figures 2, 3).

**Figure 4:**
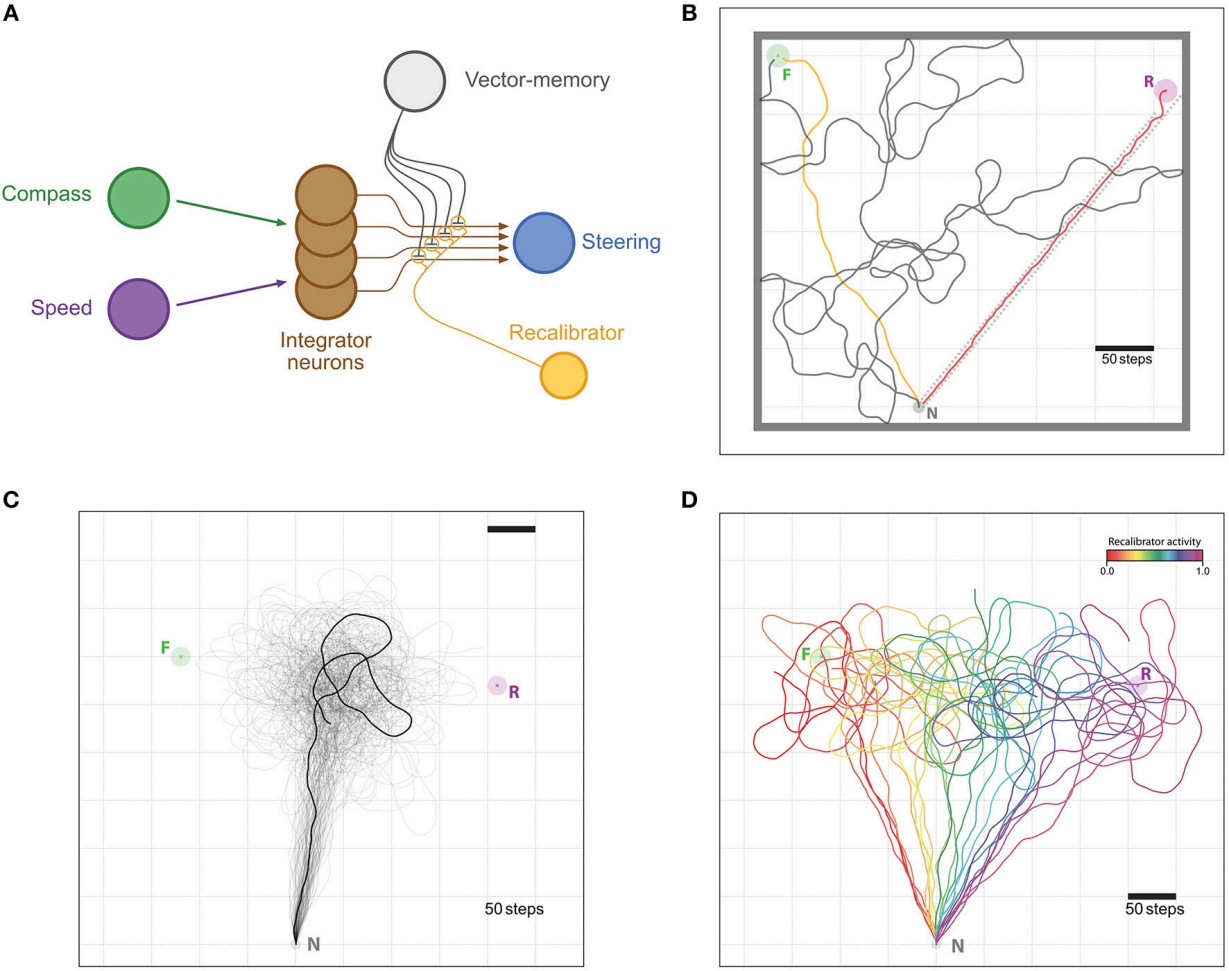
Memory re-calibration. **(A)** Same representation as in Figure 2, with the difference that synapses weights are now modulated by another neuron termed “recalibrator,” typically triggered when the agent arrives at the Nest. The weights are modulated in the opposite sign as with the “Reinforcer” neuron of Figure 2. **(B–D)** Example of the re-calibration effect. **(B)** Visualization of the training setup. The task is for the agent to leave the Nest (N, Gray circle) and find the Feeder (F, Green circle) by performing a random walk (gray trace). Once the vector-memory of the Feeder is acquired, the agent is reset to the Nest and goes out again on a memory-driven food-ward walk (Orange trace). Then, it is displaced (without any “sensory input”) to the Release site (R, Purple circle) and return to the Nest in a home-ward path (Red trace) forced by a gutter (dotted red lines). Feeder, Nest and Release site coordinates were chosen to reproduce the experimental setup in Collett et al. (1999), at scale. Thick gray lines are enclosing walls to enclose the agent for the random walk part. **(C)** Unconstrained food-ward routes. *n* = 100 individual examples (semi-transparent traces with one example more clearly marked), guided by the re-calibrated vector memory issued from **(A)** with an activity of the “recalibrator” neuron of 0.5; an averaged vector appears, replicating the food-ward paths observed by Collett et al. (1999) in ants. **(D)** Same re-calibration process, but with variable activity levels for the “recalibrator” ranging from 0.0 to 1.0 (increments by 0.05). All paths are cut at 1,000 steps.

*Vector-memory re-calibration*: Insects experiencing a PI inconsistency when returning from food to the nest due to a forced displacement, appear to make a partial adjustment of their memory of the food location (Collett et al., 1999; Wehner et al., 2002; Bolek et al., 2012) (although the extent of this “re-calibration” seems to vary with experimental conditions). We suggest how this updating of a food-ward vector-memory could occur (Figure 4).

**Figure 5:**
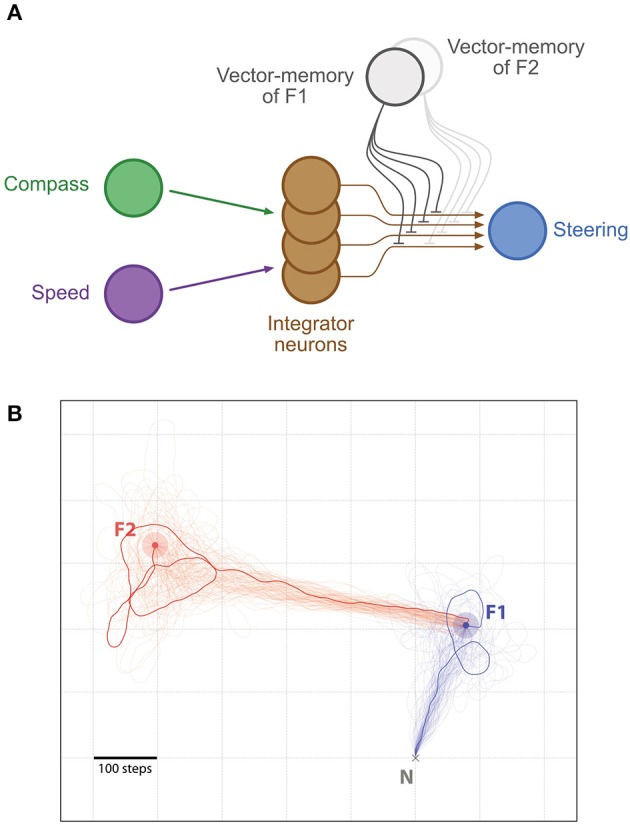
Shortcutting. **(A)** Same representation as in Figure 2, with the difference that two distinct vector-memory neurons are available (but only one recalled at a time). **(B)** Example of shortcutting: An agent walked from the Nest N to a Feeder F1 (light blue outer circle, F1 catchment area; blue inner circle, F1), under the control of the first vector-memory. Once F1 was reached, the agent recall the second vector-memory and is guided toward the Feeder F2 (light red outer circle, F2 catchment area; red inner circle, F2) by performing a shortcut (vector addition). In both segments, *n* = 100 individual paths (semi-transparent traces, with 1 more clearly marked). All paths are cut at 5,000 steps if the Feeders are not found.

*Shortcutting*: Bees have been observed to make novel shortcuts between remembered food locations (Menzel et al., 2005). It has previously been demonstrated that this can be obtained by vector addition, i.e., combining the current state of the home vector (from an arbitrary location such as a first food source) with a vector-memory from home to another food source (Cruse and Wehner, 2011). This produces a vector directly from the current location to the food. We show that such shortcutting would be a straightforward consequence of switching between memories in the CX circuit; importantly, this demonstrates how vector addition could be implemented in the insect brain (Figure 5).

**Figure 6:**
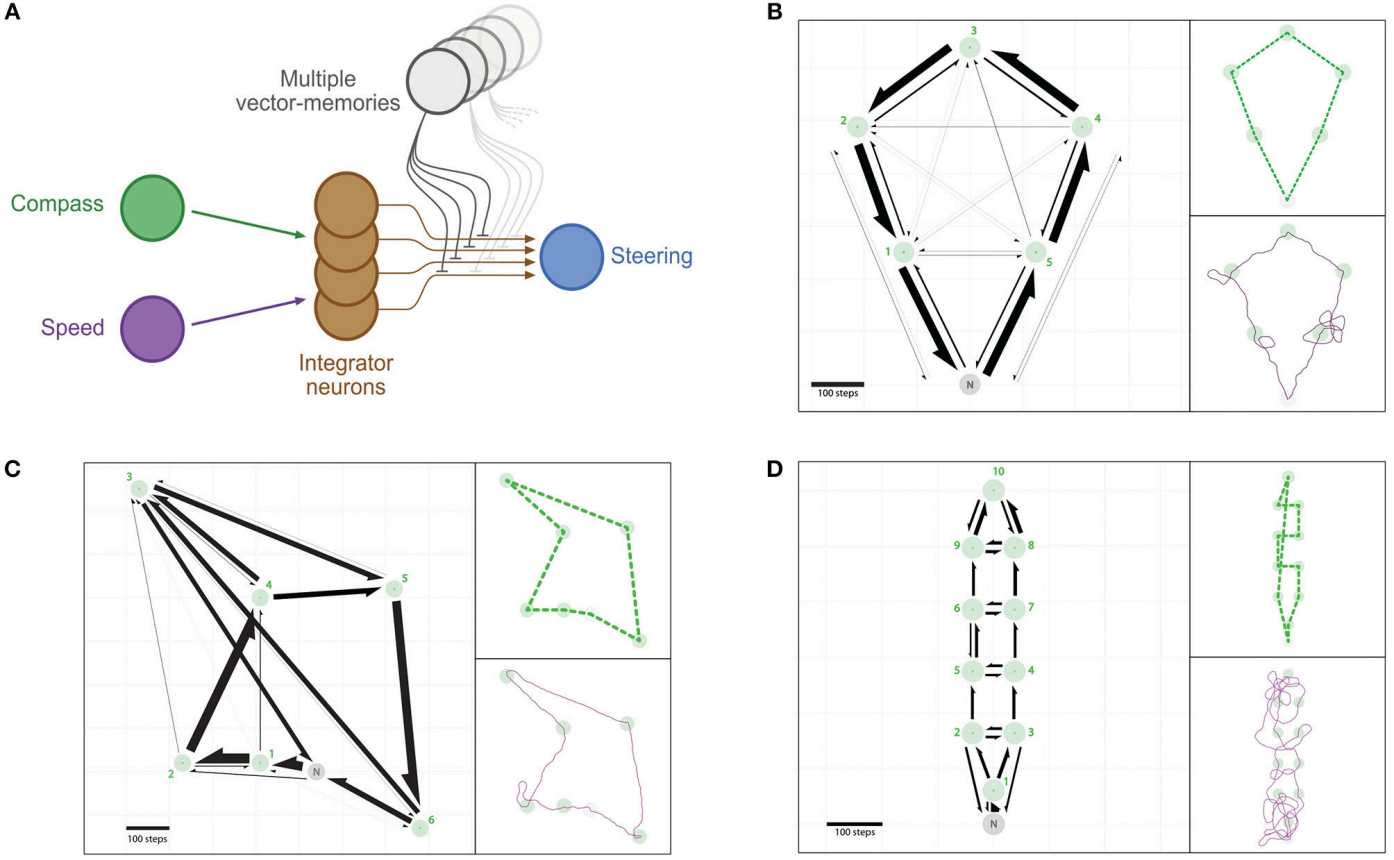
Multi-location routes. **(A)** Same representation as in Figure 2, with the difference that several distinct vector-memory neurons are available, and recalled (only one at a time) based on the selection process described in section 2.3.4. **(B–D)** Example of routes between multiple feeders across repeated outward trips: an agent having the vector-memories of all the feeders in a given array is left “foraging” thanks to a simple vector-memory selection heuristic. **(B)** Positive array (5 feeders). **(C)** Negative array (6 feeders). **(D)** Negative array (10 feeders). Left: Occurrences of direct segments between pairs of feeders represented as arrows (width is proportional to the occurrence of the corresponding segment). Green circles, feeders catchment areas; Green crosses, feeders centers; Gray circle, Nest catchment area. Top-right: Most-used route for the corresponding array. Bottom-right: Example traces for a single trip.

*Multi-location routes*: Bees often feed on multiple locations (e.g., feeders or flowers patches) before returning home, and have been shown to take efficient multi-location routes, or “traplines,” that minimize the overall journey distance (Ohashi et al., 2006; Lihoreau et al., 2012b; Buatois and Lihoreau, 2016). We investigate a simple rule by which the neural circuit output can be used to choose the next location to visit, and test whether this produces multi-location routes similar to bees (Figure 6).

**Figure 7:**
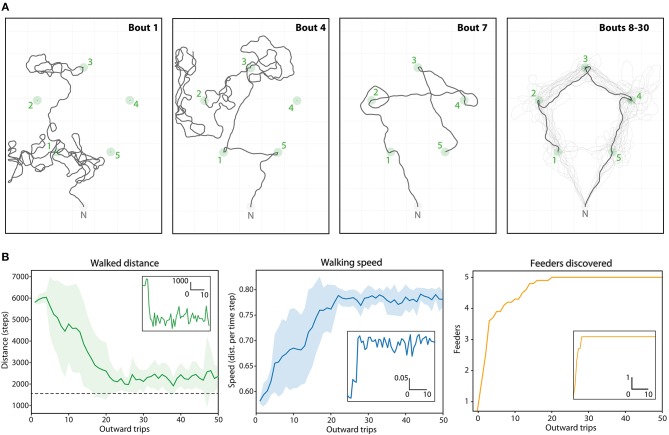
Route ontogeny in the Positive (5 feeders) array. **(A)** Example traces of one agent's outward foraging bouts over time (bouts 1->30), given the upper limit of 10,000 steps. Green circles, feeders catchment areas; Gray circle, Nest catchment area. First panel: Bout 1, two feeders discovered through random walk. Second panel: Bout 4, three feeders found by memory, and one discovered through random walk. Third panel: Bout 7, first trip where all 5 vector-memories are available immediately after leaving the nest. The route is suboptimal because the last generated vector-memory is still very noisy (feeder discovered after a long random walk). Fourth panel: Bouts 8 to 30, the trace mostly follows the optimal route, which emerges as the memories gets more precise. **(B)** Dynamics of the task (mean values over 20 repetitions) across 50 foraging bouts: distance, speed and number of feeders discovered. Corresponding insets are examples for one repetition.

*Route ontogeny*: Finally, we explore how such multi-location routes might develop over repeated foraging excursions through a combination of random exploration and vector-memory recall (Figure 7).

## 2. Methods

### 2.1. Environment and Agent

We simulate (using Python 2.7) an agent moving in a 2D environment. Movement in these simulations is discretised in time and space. Units are therefore arbitrary, and different walking “speeds” may be achieved by changing the length of the spatial step that the agent moves at a time. In the following paper, we describe the agent's movement as time steps (*t*), where the “speed” is generally kept constant during tests, but variable during random walks (see Supplementary Material section “Random Walks”). The environment typically contains a nest, one or multiple feeders, as well as optional obstacles. The nest and feeders are circular with a small defined radius (relative to the typical environment size) within which the agent is assumed to have “landed” successfully at the target, and a larger radius, or “catchment area” which is assumed to provide an olfactory signal (or other attractive signal) that could steer the agent to the target. Obstacles can have circular, rectangular or wall-like shapes and prevent the agent from passing through the area they occupy (e.g., walls enclosing the agent in an arena) by emitting a very short range repulsion signal that can steer the agent away.

The agent's size is one spatial unit. It is assumed to have sensory information about its heading direction in an absolute external reference frame, as could be supplied in real insects for example by a celestial compass (over a short time duration, or with internal clock correction, Labhart and Meyer, 2002). It is also assumed to have information about its instantaneous speed of movement in its heading direction as could be supplied by optic flow, step counting, or efference copy. These provide inputs to the CX model for path integration and control of steering. Lastly, the agent is equipped with two “detectors,” oriented at 90 degrees, that provide no input whatsoever to the neural model we describe, but only act as modulators of the agent's turning intensity in response to “attraction” or “repulsion” signals emitted by objects in the environment such as the nest, feeders, or obstacles.

The agent's starting position for each simulation is (unless specified otherwise) set at the nest. Its position is updated iteratively depending on its speed *v* and heading θ:

(1) $$\begin{array}{l} x_{t}=x_{t-1}+v_{t-1}\cos(\theta_{t-1})\\ y_{t}=y_{t-1}+v_{t-1}\sin(\theta_{t-1}) \end{array}$$

The speed and heading can be controlled by a random walk process (see “Random Walk” section in Supplementary Material) or have a fixed speed (*v*_*t*_ = 0.15) and a heading given by the outputs of the CX steering neurons (see section 2.2), depending on a flag that sets the current motivational state (see below). Or, when an obstacle or a goal is detected, the heading is given as follows:

(2) $$\begin{array}{l} M_{left}\propto (R_{left}A_{obj})\\ M_{right}\propto (R_{right}A_{obj}) \end{array}$$

(3) $$\theta =(M_{right}-M_{left})+noise$$

with *M*_*left*_ and *M*_*right*_ the modulation for left and right sides, respectively, which are proportional to the left and right readings *R*_*left*_ and *R*_*right*_ of the two detectors, multiplied by the detected object's attractiveness *A*_*obj*_. The added *noise* is drawn from a VonMises distribution centered on 0:

(4) noise~VonMises(0, κ)

where κ = 100.0 is the concentration of the VonMises distribution. Note that this is considered to be a basic reflex behavior of the agent, which by-passes the CX circuit. Finally, in such case of a environment-driven steering modulation, the agent's speed is also modulated by an increased drag value (multiplied by a factor of 1.5), providing better turns.

### 2.2. Central Complex Model

For convenience, we provide here an overview of the mathematical description of the CX model, but we deliberately omit the detailed biological justification, which is covered at length in Stone et al. (2017). Layers 1–4 are identical to the previous model. A “vector-memory” neuron has been added, which can store the output state of layer 4, and in turn, modulate this output before it reaches layer 5 (steering).

In overview, the circuit consists of a set of direction cells (layer 3) that divide the azimuthal space and are activated by the current heading of the agent (layers 1 & 2). Mutual inhibition in layer 3 forms a ring attractor circuit creating a stable distributed pattern in the form of a sinusoid. A set of integrator cells (layer 4) receive speed input but are inhibited by their corresponding direction cells and thus accumulate distance traveled opposite to the heading direction, creating a distributed representation of the home vector. The vector-memory allows the current state of the home vector to be stored when the agent is at salient locations (feeders). The state is stored in the synaptic weights of one neuron for each memory location. Homing is controlled by steering cells (layer 5) that compare the integrator cell activation to the current direction cell activation to determine if the animal should turn left or right. Vector-memory can be used to selectively influence this comparison process.

This circuit uses firing rate model neurons, in which the output firing rate *r* is a sigmoid function of the input *I*:

(5) $$r=\frac{1}{\left(1+e^{-(aI-b)}\right)}$$

where parameters *a* and *b* control the slope and offset of the sigmoid. On this value is added a Gaussian noise $N\left(0,\sigma_{r}^{2}\right)$, with σ = 0.1. This output firing rate is, across all layers, subject to a clipping between 0 and 1 to prevent the applied noise to depart from the range [0, 1]. The input *I* is given by the weighted sum of activity of neurons that synapse onto neuron *j*:

(6) $$I_{j}=\sum_{i}W_{ij}r_{i}$$

The value of the parameters for slope, offset and connection weights for each layer are provided in Supplementary Material.

#### 2.2.1. Layer 1 - Speed Input

To implement input to our speed-sensing (TN2) neurons, we simulate forward-to-backward optic flow sensing, taking into account the diagonally offset preferred angles of identified TN-cells in the CX noduli in each hemisphere (Stone et al., 2017):

(7) $$\begin{array}{l} I_{TN_{L}}=[\cos(\theta +\phi ),\sin(\theta +\phi )]\cdot v\\ I_{TN_{R}}=[\cos(\theta -\phi ),\sin(\theta -\phi )]\cdot v \end{array}$$

where **v** is the velocity vector of the agent, · the dot product, θ ∈ [0, 2π) is the current heading of the agent and ϕ is the preferred angle of a TN-neuron, i.e., the point of expansion of optic flow that evokes the biggest response. For our model, a default preferred angle of ϕ = (π/4) was used. TN2 neurons have their value clipped between 0 and 1 so that they respond in a positive linearly proportional manner to *I*_*TN*_, but have no response to negative flow (backward motion):

(8) $$r_{TN2}=\min(1,\max(0,I_{TN}))$$

In practice for this paper we assume that the agent is moving in the direction it is facing, i.e., **v** = [*cos*(θ), *sin*(θ)]*v*, which will produce an equal response in each TN2 neuron, i.e., *I*_*TN*_*L*__ = *I*_*TN*_*R*__ = cos(ϕ)*v* regardless of the heading θ.

#### 2.2.2. Layer 1 - Directional Input

The first layer of Directional input consists of 16 input neurons, each of which has a preferred direction α ∈ {0, π/4, π/2, 3π/4, π, 5π/4, 3π/2, 7π/4} with each of the 8 cardinal directions represented twice over. We identify these with polarization sensitive TL neurons in the insect central complex (Stone et al., 2017). On each time step they receive input corresponding to the cosine of the difference between their preferred heading and the agent's current heading θ ∈ [0, 2π):

(9) $$I_{TL}=\cos(\alpha -\theta )$$

#### 2.2.3. Layer 2

The second layer consists of 16 neurons that receive inhibitory input proportional to the output of the first directional input layer. This simple inversion of the response across the array is not actually crucial but is included to model the properties observed in CL1 neurons connecting the polarization input to the protocerebral bridge (Stone et al., 2017).

(10) $$I_{CL1}=-r_{TL}$$

#### 2.2.4. Layer 3 - Compass

The third layer consists of 8 neurons that get input from each pair of CL neurons that have the same directional preference. These neurons are identified with the TB1 neurons in the protocerebral bridge of the CX, which also make mutually inhibitory connections with each other in a specific pattern that resembles a ring-attractor circuit (Stone et al., 2017). Thus, their input is given by:

(11) $$I_{TB1}=W_{CL1,TB1}r_{CL1}+W_{TB1,TB1}r_{TB1}$$

where *W*_*CL*1, *TB*1_ is a [0, 1] matrix mapping pairs of CL neurons to single TB1 neurons, and *W*_*TB*1, *TB*1_ is a matrix of inhibitory weights between TB1 neurons where:

(12) $$W_{TB1_{i},TB1_{j}}=\frac{d(\cos(\alpha_{i}-\alpha_{j})-1)}{2}$$

where α_*i*_ and α_*j*_ are the preferred directions of their respective TB1 inputs, and *d* = 0.33 is a scaling factor for the relative effect of this inhibition compared to the direct CL1 excitation.

#### 2.2.5. Layer 4 - Speed Accumulation

The fourth layer consists of 16 neurons, which we associate with the CPU4 cells that occur in each column of the CX central body upper. These receive input from both the protocerebral bridge (TB1) and the noduli (TN2). The input for these neurons is an accumulation of heading of the agent, obtained by inhibitory compass modulation of the speed signal from the speed-sensitive neurons:

(13) $$I_{CPU4_{t}}=I_{CPU4_{t-1}}+acc\times (r_{TN2_{t}}-r_{TB1_{t}}-decay)$$

where *r*_*TN*2_ is the speed-sensitive response, *r*_*TB*1_ the compass-sensitive response; and *acc* = 0.0025 and *decay* = 0.1 determine the relative rates of memory accumulation and memory loss. The charge of all integrator cells starts at *I*_*CPU*_4__*t*0__ = 0.5 and, as it accumulates, is clipped on each time step to fall between 0 and 1. Note that accumulation occurs on the input, i.e., it is not affected by the non-linearity of the neuron's output function. Also note that the decay shifts the whole activity pattern toward 0, rather than moving the relative amplitude in each accumulator toward the others. As such, this does not act as a leaky integration of the path (as proposed in e.g., Sommer and Wehner, 2004 and as modeled in e.g., Vickerstaff and Di Paolo, 2005), as the relative amplitude will still encode the veridical home vector, unless the leak (or the accumulation) are enough to cause the values to be clipped at 0 (or 1). The 8 TB1 neurons each provide input to two CPU4 neurons which will thus have identical activity (other than added random noise, see below) as we assume the agent moves in its heading direction thus generating symmetric optic flow. As these neurons integrate the velocity (i.e., speed and direction) of the agent, the activity across this layer at any point in time provides a population encoding of the home vector.

#### 2.2.6. Vector-Memory

This is the only new component in circuit compared to Stone et al. (2017). It is a hypothetical addition and as yet we do not suggest any specific identified neural analog. We store the vector-memory in the synaptic weights of a hypothetical memory neuron that inhibits the output of the CPU4 integrator cells: i.e., the memory neuron has 16 inhibitory output synapses, one per CPU4 output fiber (see Figures 1B, 2A).

The weight of these synapses are set according to the corresponding activity of the CPU4 output fiber at the moment of learning, as could be signaled by a reinforcer neuron. More precisely, we store the *I*_*CPU*4_ values after passing through a sigmoid function of the same slope and bias parameters as the CPU4 response (see Supplementary Material, “Neurons parameters”), but without any added noise. This is to avoid encoding the instantaneous noise level (i.e., the one of the last time step only), and can be interpreted as the learning taking place over a short time interval to more precisely estimate the current CPU4 activity. The noise is then added dynamically (at each time step) during recall, like in the rest of the system. The obtained values are negated in sign (since the synapses are inhibitory). In other words, the agent's current home vector gets stored in the 16 synaptic weights of the memory neuron when the reinforcer neuron is triggered (Figure 1D). The learning of the vector-memories is set at particular time or locations: in this paper, these are associated with the discovery of food. As described below, this will allow the agent to return to the position at which the vector was stored. For some experiments we allow the agent to store more than one such vector-memory, into separate memory neurons, corresponding to different food locations.

Thus, the vector-memory synapses can be represented as a 16-values vector *W*_*VM*_:

(14) $$W_{VM}=-\{\begin{array}{l} r_{CPU4_{noiseless}},\\ baseline, \end{array}\;\begin{array}{l} \text{if signaled to store}\\ \text{otherwise} \end{array}$$

with *baseline* being a vector of 16 zero-state values (= 0.5, since firing rate is encoded between 0 and 1).

#### 2.2.7. Vector-Memory Recalibration

We also introduce a potential re-calibration of the vector-memories, based on the state of Layer 4 when the agent reaches the nest. In the absence of error (either noise or induced through an experimental manipulation) this state should be zero, so any remaining activation in the Layer 4 thus encodes a possible “error vector” accumulated across the whole path (inbound and/or outbound).

This “error vector” can be used to modulate the vector-memory synapses. For this, another hypothetical process very similar to the learning described above, is used: a “recalibrator” neuron, triggered when the agent arrives at the nest, modulates the vector-memory synapses that were last active, similarly to the reinforcer neuron used for learning, only differing in the sign of the modulation. That way, the potential “error vector” remaining in the CPU4 population causes the re-calibration of the last active vector-memory (Figure 4A).

Thus, the vector-memory *W*_*VM*_ update:

(15) $$W_{VM_{rec}}=W_{VM}+r_{rec}(b-r_{CPU4_{N}})$$

where baseline *b* = 0.5, *r*_*CPU*_4__*N*__ is the output of the integrator when the Nest is reached, *r*_*rec*_ is the activation of the “recalibrator” neuron, or in other words the efficiency of this re-calibration. For instance, with an efficiency *r*_*rec*_ = 1, the updated vector-memory will be fully corrected for the error. For *r*_*rec*_ = 0.5 the result will be an average between the previously stored vector-memory and a fully error-corrected one.

#### 2.2.8. Layer 5 - Steering Output

This layer contains 16 neurons which receive input from the compass (layer 3), and the home vector (layer 4) modulated by the vector-memory neuron. These inputs can be switched on or off depending on the agent's state, e.g., whether it is attempting to return home or to return to the location where a vector was stored. The input from the compass layer 3 is inhibitory, following the same pattern as the layer 3 to layer 4 connections. The connections from layer 4 to layer 5 are offset, by one column to the left for one set of 8 neurons *CPU*1_*L*_, and by one column to the right for the other set of 8 neurons *CPU*1_*R*_. The vector-memory synapses modulate the output from layer 4 to layer 5.

We identify the steering neurons with the CPU1 neurons in the central body upper of the CX, which anatomically reveal the offset pattern used in the model. Inside layer 5 are also pontine neurons that receive the same pattern of input from layer 4, and provide inhibitory output that balances and filters the activity across both hemispheres (see Stone et al., 2017 for more detail). For convenience we neglect the pontine neurons in the equation below because they do not affect the circuit when using symmetric speed input:

(16) $$I_{CPU_{1}}=\{\begin{array}{l} W_{TB1,CPU1}r_{TB1},\\ W_{TB1,CPU1}r_{TB1}+W_{CPU4,CPU1}r_{CPU4},\\ W_{TB1,CPU1}r_{TB1}+W_{CPU4,CPU1}r_{CPU4}+W_{VM}r_{VM} \end{array}\;\begin{array}{l} \text{when exploring}\\ \text{when homing}\\ \text{using vector-memory} \end{array}$$

where *W*_*CPU*4, *CPU*1_ is the connectivity matrix from CPU4 to CPU1 cells, *W*_*VM*_ is synapses weight vector of the vector-memory and *r*_*VM*_ is the activation of a specific vector-memory neuron (basically *r*_*VM*_ = 1 when using that vector-memory, *r*_*VM*_ = 0 otherwise).

The output of CPU1 cells project to the left and right lateral accessory lobes, which are pre-motor centers. We thus use the difference in *CPU*1_*L*_ and *CPU*1_*R*_ sets to provide a steering signal for the agent:

(17) $$\theta_{t}=\theta_{t-1}+0.5(\sum_{i=1}^{8}r_{{\text{CPU1}}_{Li}}-\sum_{i=1}^{8}r_{{\text{CPU1}}_{Ri}})$$

Note first that in the “exploring” state, the left and right activity will be identical and hence will not affect the steering. In the “homing” state, the circuit effectively performs a comparison of the population vectors representing current heading (compass) (*TB*1) and the integrator *CPU*4, but the connectivity pattern between the integrator and the steering cells means that the desired heading signal is offset in both directions by one column. Hence the left and right activity of the steering cells will represent whether the left or right offset provides a better alignment, and the difference between them can be used to steer, as described in Equation (17). As the integrator keeps running, the steering signal will disappear (or be dominated by noise) when the agent nears home, producing a search pattern.

In the “using vector-memory” state, the output of the integrator is balanced by inhibition from a vector-memory stored at a feeder location (see above). If starting from the nest, with the integrator containing a zero home vector, this negative influence means the agent acts as though its own location (for the purpose of steering) is exactly opposite to where the feeder is located, and the steering circuit will drive it “home” from its actual location (the nest) toward the food. Since the path integration continues to run in parallel, accurately reflecting the agent's actual displacement, when the food location is reached the input from the integrator to the steering layer will cancel out the negative influence from the vector-memory and the agent will start its search pattern, just as it would at the end of a regular “homing” state.

### 2.3. Experimental Paradigms

#### 2.3.1. Memory-Directed Movement

To observe the efficiency of the memory-directed movements, the task is realized in two parts: First, the agent performed random walks of different lengths, originating from the nest (*x* = 0, *y* = 0), and stored for each of these the final integrator state as a new vector-memory. Then, after being reset to the nest (coordinates reset to *x* = 0, *y* = 0; integrator reset to baseline = 0.5), a vector-memory was recalled and allowed to drive the behavior. We used a feeder catchment area of 20-steps radius: as soon as the agent entered the feeder catchment area, its proximity sensors guided it to the feeder location. We typically ran *N* = 1, 000 trials at 20 random-walk lengths, equally spaced between 100 and 10,000 steps.

A basic measure used was the proportion of successful trials. We considered a food-ward route successful if the agent reached the feeder coordinates within a given time limit of 5,000 steps. It is expected that the agent reaches the target in a straighter path and then performs random search around the expected location. We also evaluated the systematic search patterns produced, either by an agent returning home after a random walk, or an agent using a vector-memory from the nest location to return to the food (see “Systematic search” section in Supplementary Material). In this case, there was no actual nest or feeder object (or associated catchment area) and instead we allowed the search to continue for 10,000 steps.

#### 2.3.2. Memory Re-calibration

We tested the idea of a vector-memory recalibration in simulated open-jaw experiments, by forcing an incongruity between the outbound and the inbound routes similarly to the experiments of Collett et al. (1999) with ants, and Otto (1959) with bees.

In this task, the agent had first to discover a single feeder location by performing a random walk from its nest in an enclosed area to generate the corresponding vector-memory. Subsequently, we let the agent travel again from the nest to the goal location using its vector-memory. Once this was successfully achieved, we simulated a passive displacement by instantaneously changing its coordinates to a novel release location. We then forced the agent's path back to the nest by using wall obstacles disposed in a gutter-like arrangement (see Figure 4B). When the agent reached the nest, its integrator would have recorded the forced displacement but not the passive displacement and will therefore not be at the zero-state. The error vector thus encoded was used to make a correction in the vector-memory as described in section 2.2.

The re-calibrated vector-memory was then used in the test task, for *N* = 100 repetitions. We recorded the paths taken for the averaged re-calibration (efficiency *r*_*rec*_ = 0.5), as well as for 10 different values of efficiency. Note that since we only forced an error during the inbound part, this re-calibration becomes a direct way to change the relative weight of the outbound and inbound routes.

#### 2.3.3. Shortcutting

At any point in a vector-memory enabled walk, the agent is driven by the combined effect of the recalled vector-memory and the current home vector. The agent will try to “home” to the location where these are balanced, even if it is forced to take a detour, or has previously moved by itself to another location (e.g., using the vector-memory of a different feeder). Effectively, this constitutes the subtraction of two vectors: one directed from the agent's current location to the nest, and the second directed from the target feeder location toward the nest, so that its behavior follows the vector between their end-points. In other words, the agent should take a direct shortcut to the second food source.

In our shortcutting experiment, the agent first had to discover independently two feeders, by performing two independent random walks (being reset at the nest in-between these walks), storing the two corresponding vector-memories. Then, it used one of these two memories to go back to the associated feeder as described above in the section 2.3.1 experiment. If the first goal is reached, the inhibition from this memory is lifted and the second vector-memory is activated. We evaluated the success rate in reaching the second goal, the path straightness during the shortcut, and the angular error when leaving the first feeder.

As in the section 2.3.1 experiment, we generated a large set of vector-memories, by launching sequentially 1,000 outbound random walks, of length varying between 100 and 10, 000 steps, binned in 20 equally spaced intervals (i.e., 50 independent random walks per length). We then drew *N* = 1, 000 couples of feeders from this bank so that the straight-line distance between the two feeders ranged between 100 and 2, 000 steps, binned in 20 equally spaced intervals (i.e., 50 independent repetitions for each of the 20 distances bins), while making sure that the Nest - Feeder 1 distance was as uniformly distributed as possible.

#### 2.3.4. Multi-Location Routes

In our multi-location routes experiments, the agent had as a task to take a multi-feeder route, based on a bank of previously stored vector-memories, before going back to the nest.

The order of feeder visits is based on the fact that the distance between the current location and a given memory location can be obtained from the input to the steering cells after inhibition by a specific vector memory (i.e., the subtraction of the 16 synapse weight values from the 16 CPU4 values). The amplitude of the sinusoidal signal across the 16 values directly correlates with the distance between current and memory location. We used an approximation that would be simple to obtain neurally: the sum of the CPU4 activation values after the subtraction of a given vector memory. Note that alternative approximations for the relative distance could be used, such as the value of the cell that is the most active among the 16 cells.

Given *k* vector-memories, if each is subtracted in turn from the current integrator state *r*_*CPU*4_, then for each we can define a global activity value *Score*_*k*_ (after clipping the resulting activity between 0 and 1):

(18) $$Score_{k}=\sum_{i=1}^{16}\left(r_{CPU4_{t_{i}}}-r_{VM_{k_{i}}}\right)$$

The agent selects the vector-memory generating the smallest *Score*_*k*_ and sets it as the current vector-memory to drive behavior. However, the scoring process is carried out continuously, so at any time it might change to another vector-memory if its score happens to be lower than the current active one. If the agent reaches a feeder at the vector-memory location, it marks that vector as unavailable for recall for the remainder of the trip. Once no vector-memories are available, it will automatically follow its current PI to go home.

We tested this task in three different feeders arrays: a pentagonal array with 5 feeders where nearest neighbor and the optimal routes are equivalent (Lihoreau et al., 2012b), an array with 6 feeders where the nearest neighbor and the optimal route differ (Lihoreau et al., 2012a) in which real bees were found to select the optimal route, and another array with 10 feeders (Ohashi et al., 2006) but in which real bees were not found to select the optimal route.

To see what sequence of feeder visits would emerge for an agent highly familiar with these arrays, we first allowed the agent to discover and store a vector for each feeder in multiple random walks, repeated for an arbitrary high number of discoveries (at least 100 discoveries per feeder). We then averaged the 100 discoveries to obtain a highly accurate vector-memory for each feeder. Then in the tests, an outward trip corresponds to an agent leaving the nest, exploring or following its memories, and going back to the nest either once all feeders have been found or once a time limit is reached. One trial consists of 50 of these outward trips.

To evaluate performance, we looked at the geometry of the routes the agent realized over 500 repeated trials. The success rate was determined by the number of trials where the agent found all feeders and returned to the nest. Considering only the successful trials, we looked at the sequence of feeder visits, on full routes (occurrence of each possible route connecting all the feeders), as well as at individual feeder-to-feeder moves.

To this end, we only logged the actual visit orders and not the vector-memory recall processes. That is to say, if an agent located on feeder A recalled say, vector-memory of feeder B, but actually missed feeder B and found feeder C instead, we counted this as a path from A to C. Revisits to a same feeder were excluded (as per the bee data, e.g., Lihoreau et al., 2012a,b) by making feeders “disappear” from the agent's detection once they had been visited.

#### 2.3.5. Routes Ontogeny

In order to demonstrate that a route could emerge without necessarily needing the accurate memories used in the previous section, we performed the following experiment on the pentagonal array (Lihoreau et al., 2012b) with a naive agent (without prior knowledge of feeders locations), that gradually learned new food locations through random discovery, while also visiting any locations already learnt:

We here used feeders containing a food amount, and an agent that was assumed to have a crop equal to the sum of all feeders' food (i.e., the agent could only be fully fed after having visited all the feeders). The agent leaves the nest in a naive state, as it does not possess any vector-memory of the feeders in the test environment. The rule is to use vector-memories if any are available, by recalling them using the previously described process, and if no vector-memory is available, perform a random walk until a feeder is found. We also fix a time limit of 10,000 steps, to prevent any saturation that may occur with longer random walks. When a feeder containing food is discovered through random walk, a new vector-memory is created; if a vector-memory is currently active when a feeder is found, this memory is updated (replaced) by the current integrator state. In both cases this updated/newly created vector-memory is not made available to recall until after returning to the nest. As with the traplining experiment, the agent returns to the nest only once all feeders have been visited or when the time limit has been reached.

We observed the change in the duration of the outward trips, the change in total distance walked, and the evolution of the visit sequences. Additionally, we looked at the amount of outward trips needed to visit all the feeders, and to visit all the feeders using the optimal route. Note that once all feeders have been visited, the subsequent trips will be equivalent to those in the section 2.3.4, although memories should gradually become more accurate.

## 3. Results

### 3.1. Memory-Directed Movement

We looked here whether the agent could return from the nest to a location it had reached at the end of a random walk. The agent stored a vector memory at this location, which can be dubbed “feeder location.” We tested 20 random walk distances spanning between 100 and 10, 000 steps, with 50 trials per walking distance. To make sure the neurons are not saturating (see Supplementary Material section “Saturation” and Figure S3), we only used the random walks that ended in a radius of 700 steps from the nest for analysis.

We investigated first the homing performance, by looking whether the agent could home (i.e., reach the nest) from the feeder location. Given an upper limit of 5, 000 steps, the success for the homing task was of 100% (0 out 827 trials failed). We then investigated the ability of the agent to return to the feeder location from the nest, using its vector memory. Given an upper limit of 5, 000 steps, the rate of success in returning to the feeder location was 93.71% (52 out of 827 trials failed). The paths were rather straight (Figures 2, 3), with a straightness index (i.e., beeline/walking distance) of 0.90 for homing and 0.85 for returning to the feeder (which is significantly different for *n* = 790: paired *t*-test *t* = 5.322, *p* < 0.001). For an analysis of the precision and accuracy of our model in finding the goal, see Supplementary Material: Path analysis.

### 3.2. Memory Re-calibration

We aimed here at capturing the ability of insects to recalibrate the outbound vector-memory based on their last inbound run, which we tested by displacing an insect and forcing a homing route that produces a large outbound-inbound discrepancy, as experimentally achieved in ants (Collett et al., 1999). Over 100 subsequent outward trips, the re-calibrated outward paths resemble closely those of real ants. That is, the agent aims at a location that lies in between the two experimental ones: roughly averaging the distance and direction of the previous outbound and inbound paths (Figure 4C).

Other studies showed that ants may weight the previous outbound trip more than the inbound trip (Wehner et al., 2002), or even do not recalibrate at all (Wehner and Flatt, 1972). Since the error we introduce is only during the inbound trip, we were able to reproduce these differential weightings of the outbound and inbound trips by varying how much the synaptic weights of the vector-memory neuron are modulated by the PI state during re-calibration: from paths aiming at the feeder for weak synaptic change to path aiming at the release location for strong synaptic change overriding the previous memory (Figure 4D).

### 3.3. Shortcutting

We tested whether vector-memories could be used to realize novel shortcuts between two known locations. Here the agent has stored two goals as vector-memories, discovered independently. To test for shortcutting, the agent at the nest recalled the memory of a first feeder and, once arrived at this goal, recalled the memory of the second feeder. We observed whether the agent was able to strike a direct path between the two feeders (Figure 5). Here again, to prevent saturation of the neurons (see Supplementary Material section “Saturation” and Figure S3) we only considered trials where both feeders were within the radius of 700 steps of the nest. Also, we considered only the agents that successfully reached the first feeder (193 out of 212 individuals).

Given a upper limit of 5, 000 steps, the rate of success in reaching the second feeder from the first feeder was around 89.6% (20 out of 193 individuals failed to reach Feeder 2 from Feeder 1). We carried an analysis of the directional and positional error of the shortcuts displayed by systematically varying the spatial relationship between the nest and the feeders (see “Shortcutting: Error analysis,” in Supplementary Material).

### 3.4. Multi-Location Routes

We tested whether a route could emerge assuming the agent had memorized multiple feeder locations. In this section, the agent already possesses a vector-memory for each feeder location, and the memories do not change over trials. We use a simple heuristic to decide which vector-memory to recall: the agent recalls the memory that yields the weakest overall output activation after subtraction to the current PI state. We tested three different feeder arrays from the bee literature. For each array, we launched 500 independent trials and observed the sequences of feeders visited within a time limit of *T* = 10, 000 steps (+*T*_*h*_ = 2, 500 steps for homing).

#### 3.4.1. Positive Array (5 Feeders)

We found that 94.20% (*r* = 471) of all trials were successful in the sense that all 5 feeders had been visited and the agent went back to the nest before the time limit (Figure 6B). There are !5 = 120 possible routes to visit the 5 feeders in this array. We found that, respectively, 77.71% (*r* = 366) and 15.07% (*r* = 71) of the trials used the two optimal routes (anti-clockwise and clockwise; 5, 4, 3, 2, 1 and 1, 2, 3, 4, 5, respectively); both cases totalling 92.78% (*r* = 437) of trials. The sub-optimal nearest-neighbor routes (1, 5, 4, 3, 2 and 5, 1, 2, 3, 4) were used only in 1.49% (*r* = 7) and 0.64% (*r* = 3), respectively. Two other routes were used in less than 2% of trials, and 6 other routes were used in less than 1% of trials. The other 108 possible routes to join the 5 feeders were never used (see Supplementary Table 2 for details).

The overall distribution of direct segments effected between pairs of feeders resembles closely that observed in real bees tested in a similar feeder configuration (Figure 6B, Supplementary Table 1).

#### 3.4.2. Negative Array (6 Feeders)

In this second array, 94.00% (*r* = 470) of all trials were successful. There are !6 = 720 possible routes to visit the 6 feeders of this array (Figure 6C). Here, only 2.77% (*r* = 13) of the trials used the optimal route (1, 2, 3, 4, 5, 6). However, we found that 47.23% (*r* = 222) of the trials used the second to optimal route (1, 2, 4, 3, 5, 6). This route can be described as “suboptimal” in the sense where it is not the shortest, but it is still better than the nearest-neighbor route (1, 2, 4, 5, 6, 3), which has been used in 41.28% (*r* = 194) of the trials. 2 other routes (2, 1, 4, 5, 6, 3 and 2, 1, 4, 3, 5, 6) were used in, respectively, 3.62% (*r* = 17) and 3.40% (*r* = 16) of trials, and 4 other routes were used in less than 1% of trials. The other 711 possible routes to visit all 6 feeders were never used (see Supplementary Table 1 for details).

The overall distribution of direct segments effected between pairs of feeders differs from that observed in bees in this similar feeder configuration. This difference arose mostly because the agents did not perform a direct segment between flowers 2 and 3 as often as the bees did (Figure 6C), which we discuss later.

#### 3.4.3. Negative Array (10 Feeders)

In this third array, 95.40% (*r* = 477) of all trials were successful. There are !10 = 3, 628, 800 possible routes to visit the 10 feeders of this array (Figure 6D). The agent explored a much larger number of different routes (371) than in the previous arrays (12 and 9). No preferred route emerged here, the most used route was displayed in only 2.31% of trials. The four most used routes are not optimal in length nor do they correspond to the nearest-neighbor ones (see Supplementary Table 3 for details), even though they are closer to the latter. The three next preferred route correspond to optimal routes (clockwise and anti-clockwise rotations, either passing through feeder 1 first, or last), and these were used in a total of only 1.05% (*r* = 5) of trials. 364 other routes have been used in less than 1% of trials each. The other 3,628,429 possible routes have never been used.

This third array appears to be strongly dependent on stochasticity. This is probably due to a combination of two factors: the short distance between feeders yielding stronger directional inaccuracies (Figure 6C, and Supplementary Table 3); and the similar distance between different feeders options increases the stochasticity of the recall.

### 3.5. Routes Ontogeny

We used the positive pentagonal array to test whether such efficient multi-location routes could emerge using a naive agent that needs first to discover the different feeders through random walks (Figure 7A). Each time the agent discover a feeder, it stores a new vector-memory that will be available for the next trips. The agent was recorded over 50 successive trips. In each trip, the agent would “home” either after a limit of 10,000 steps or if it has visited all the flower locations (i.e., assuming is crop capacity is filled). Over 20 repetitions of such 50 trips' ontogeny, the variation and dynamics resembled that of bees in a similar task. The median amount of number trips needed to find all feeders was 12 (*min* = 3, *max* = 20), and the median number of trips needed to realize an optimal route was 13 (*min* = 5, *max* = 21). Interestingly, the optimal route did not necessarily emerge as soon as the 5 feeders were discovered, but was achieved within 0 to 2 trips after. This is because some memories can be at first very noisy due to the long random walks that led to their discovery. Across trials, the memories becomes more precise as the agent reaches the feeders more straightforwardly, and the optimal route eventually emerges (Figure 7A).

The overall travel distance decreases steadily until reaching a plateau between 20 and 25 trips, close to the shortest straight-line distance. Mean traveling speed increases in a similar dynamic, as fewer turns and straighter segments implies faster movements (Figure 7B).

## 4. Discussion

Insects such as ants and bees are known to use Path Integration (PI) to return in a straight line to their nest (Müller and Wehner, 1988; Collett and Collett, 2000b; Wehner and Srinivasan, 2003), but also store vector-memories to return to a previously experienced location where they have found food (Wehner et al., 1983; Collett et al., 1999; Wolf and Wehner, 2000). These vector-memories can potentially support additional behaviors such as direct shortcuts between food locations, as shown in previous theoretical models (Cruse and Wehner, 2011). Here we demonstrate that a variety of vector-based navigation behaviors can be obtained from simple extensions to a PI model which follows the anatomical connectivity of the central complex (CX) (Stone et al., 2017).

### 4.1. Vector-Memories and Novel Shortcuts

The key to the functioning of the model is that, during homing, the steering layer of the CX network continuously compares the distributed encoding of the current heading to a left or right rotation of the distributed encoding of the PI state (the desired heading). This produces an appropriate left or right turn signal to reduce the difference, resulting in a relatively straight path home, at which point the PI state is balanced. In the extended model presented here, the effect of the PI state on steering can be modulated by inhibition from a vector-memory (Figure 1B). The balance point will now be the location where the vector-memory was stored (Figure 1C), so the same steering circuit produces a direct path to food (Figure 2), as observed in insects (Wehner et al., 1983; Schmid-Hempel and Schmid-Hempel, 1984; Collett et al., 1999; Wolf and Wehner, 2000). Removing the inhibitory effects of memory, once the target location is reached, allows steering by the PI state back home again. Alternatively, switching to inhibition by a different vector-memory produces a direct shortcut from the current location to the next goal (Figure 5), as observed in bees (Menzel et al., 2005). As for homing, this steering is robust to any imposed deviation from the intended route (Wehner and Srinivasan, 2003). The way vector-memories are compared to the PI state, and can be selected sequentially to produce shortcuts, is functionally equivalent to former models based on Cartesian vectors (Cruse and Wehner, 2011; Hoinville et al., 2012; Hoinville and Wehner, 2018) but in the present paper it is done with a neurally more plausible ring-neuron representation of vectors.

### 4.2. Dealing With Inaccuracies

Any PI mechanism necessarily accumulates errors (Cheng et al., 1999; Wehner and Srinivasan, 2003), raising the issue of how insects might deal with such errors. If they do not find the goal, whether home or a food source, insect display a systematic search for it (Fourcassié and Traniello, 1994; Merkle and Wehner, 2009; Schultheiss and Cheng, 2012; Wolf et al., 2012). Similarly, the proposed CX model spontaneously results in a search around the expected goal location (Figure 2), as in the original model for homing (Stone et al., 2017) and as well as in another model (Hoinville and Wehner, 2018), suggesting that systematic search may not require an additional “search module,” as often assumed (Wehner, 2009; Cruse and Wehner, 2011; Wystrach et al., 2013).

The question of PI errors also raises the question of whether and how insects might recalibrate their memories. We introduced two mechanisms by which a vector memory might become more accurate. The first follows from the analysis above—there will be less error in the PI state if the animal reaches a food location on a more direct path from the nest, so increasing precision can be obtained by updating the “active” vector-memory, when the goal is reached, with the current PI value, as we observe in route ontogeny (Figure 7A).

There is some evidence in insects of a second mechanism. Manipulating the return path from a food source to the nest can affect the vector-memory (Otto, 1959; Collett et al., 1999; Bolek et al., 2012). We showed how this could be effected in our CX model by allowing the vector memory stored at a goal location (the set of weights) to be adjusted, when the agent has reached home, proportionally to the remaining PI signal, which denotes accumulated errors. This recalibration simply requires the same assumed synaptic connectivity than for learning a vector-memory at the first place (Figure 4A). It only implies a second instant in which synaptic weights are altered, rather than an independent PI system for outbound vs. inbound routes. Note that this adjustment could be done simultaneously for all memories either formed or activated on the most recent journey.

In insects, the influence of the homeward path on the next outbound paths varies across experiments (Wehner et al., 2002; Menzel and Greggers, 2015), or sometimes seems non-existent (Wehner and Flatt, 1972). In our model, such variation can be achieved by changing the strength of the synaptic modulation applied during recalibration (Figure 4). This effectively results in using different proportions of the PI error when making this adjustment (Figure 4D). It remains unclear whether these differences result from differences in species, motivational state, environmental circumstances or individual experience.

Of the “memory neuron” accordingly to the remaining activity of the neurons onto which they synapse. That is, similarly to the way we suggest vector-memory are learnt in the first place, excepted that the synaptic modulation is in the opposite direction, and should happen once the agent has reached home.

### 4.3. Multi-Feeder Routes

We further extended the shortcut process to explain the development and maintenance of efficient routes between multiple feeders as exhibited by bees (Ohashi et al., 2006; Lihoreau et al., 2012b; Buatois and Lihoreau, 2016). This required two assumptions: 1-the agent needs to select one vector-memory at a time, and 2-a memory becomes unavailable once that location has been visited. We implemented a simple continuous memory selection mechanism, as has been previously proposed (Hoinville et al., 2012). To do so, we used the fact that, in the CX circuit, the inhibition of a target vector-memory onto the PI results in activation levels which amplitude is proportional to the distance to be traveled (Figure 1C). At each time step, the current vector-memory recalled can thus be the one that results in the smallest amplitude. Several proxies could be used to approximate this amplitude, but how this is implemented neurally remain to be seen. This produced multi-location routes in our agent that are surprisingly similar to that of bees (Figure 6), including the discovery of optimal (shortest possible) routes for some feeder arrays (Lihoreau et al., 2012b), and less optimal routes for other layouts (Ohashi et al., 2006; Woodgate et al., 2017). Alternative hypotheses for memory-selection could exist, but a continuously running winner-take-all mechanism seems parsimonious and readily testable: for example, by enforcing a detour toward a feeder B to a bee on its way to a feeder A and looking for an eventual motivational switch from A to B.

Different ways of storing and selecting vector memories might result in slightly different multi-feeder route outcomes, but the key point is that bees would not need to store, nor compare any additional information (such as path length) about previous journeys to be able to improve their performance over time. Importantly, in this model such multi-feeder routes do emerge, no matter the memory selection mechanism, and without the need to make a comparison of the total traveled distances across successive paths, which was assumed in previous theoretical models (Lihoreau et al., 2012b; Reynolds et al., 2013).

Note that in one of the arrays, the preferred route adopted by our model was not the preferred route of the real bees, but their second preferred one (Figure 6C). However, insects do not rely only on vector based strategies, and additional mechanisms, such as the use of terrestrial cues, are likely to modulate the way they follow routes. Spontaneous bias may also influence the shape of a route. For instance, bumblebees have a natural tendency to depart from a flower in the same direction as they arrived (Pyke and Cartar, 1992), which we did not implement here.

Finally, our model could also produce a realistic ontogeny of such multi-feeder routes (note however that we tried here only the regular pentagonal array), given the simple assumption that an agent with no vector-memory available to recall triggers a random walk (Figure 7A). In this case vector-memories are gradually added as the agent discovers new flowers. As a consequence, paths become straighter and the revisits order becomes more efficient across successive trips (Figure 7A). Interestingly, the ontogeny dynamics of our agents in the pentagon array (Figure 7B) resembles that of real bees (see Supplementary Material for more details).

### 4.4. Insights Into Behavior?

Our study thus shows that for direct return to a goal, search around the goal location, shortcuts between goals and efficient route discovery between multiple goals, vector manipulation is a highly parsimonious explanation for observed insect behavior because it appears strongly consistent with the known architecture, and likely computational function, of the CX.

Can our proposed CX implementation however provide predictions about systematic errors in insects, over and above that which has already been provided by canonical PI models (Cheung and Vickerstaff, 2010; Vickerstaff and Cheung, 2010; Cheung, 2014; Hoinville and Wehner, 2018)? We note that the effective PI calculation carried out by our CX circuit model is equivalent to an allocentric Cartesian encoding, and as such, theoretical results concerning the effects of sensory or internal noise on accuracy and precision in return to home or a vector goal derived from mathematical models of this form (Cheung and Vickerstaff, 2010; Cheung, 2014; Hoinville and Wehner, 2018) should apply. This is broadly true for our simulation (see detailed analysis in Supplementary Material). For example, we find that directional precision (perhaps counterintuitively) increases with nest-feeder distance, for both inbound and outbound paths, and does not depend on the length of the random walk made before discovery of the feeder, which is consistent with both canonical PI models (Hoinville and Wehner, 2018) and results in ants (Wystrach et al., 2015).

However, we note that observed error effects may be dependent on particular, and somewhat arbitrary, choices in our neural and/or behavioral modeling. For instance, we believe the non-linear activation function of neurons used in the model may explain some of the errors observed, such as an underestimation of distance (see Supplementary Material). It is also possible that some of our results are a consequence of (equally arbitrary) parameters in our random walk model (Cheung, 2014). Examination of the consequences of varying these choices would be interesting but is beyond the scope of this paper, which aims to provide a proof-of-principle, rather than provide strong quantitative predictions about animal behavior. However, one general outcome that should hold is that errors for foodward routes should always be higher on average than for homeward routes, as observed here (Figure 3), because the control depends on both the current noise in PI and the noise in the vector-memory, from the PI state when it was stored. As the focus of this paper was to show an “in principle” mechanism for vector memory in the insect brain, we leave more detailed examination of how parameter choices in the CX model might affect errors to future work.

### 4.5. Insights Into Neural Circuits

It is of interest to consider whether the neurobiological assumptions made in our model could be verified:

- We modeled vector-memory as simple storage of a copy of the 16 discrete values in the CPU4 layer that represent the home vector at that point in time. We suggest that a vector-memory could be encoded by a single “vector-memory neuron” that sends inhibitory connections to the output of all the integrator neurons (Figure 2A). We therefore suggest the existence of such inhibitory neuron projecting to all wedges of the CPU4 outputs or analogous CX layers that would also encode current PI state. Note that similar global inhibitor neurons have been evidenced in drosophila (Kim et al., 2017).
- Learning a vector-memory would therefore consist in setting the weights of such inhibitory connections. Each output synapse of the vector-memory neuron should be weighted according to the neural activity of CPU4 neuron onto which it synapses, when at the feeder. Such synaptic modulation could be achieved by a reinforcer neuron triggered by the food intake at the feeder (Figure 4A). Likely candidates are dopaminergic (Kong et al., 2010) or octopaminargic (Wolff and Rubin, 2018) neurons that are known to project into the central complex.
- Re-calibration would consist in modulating the output synapses of a learnt vector-memory neuron. As for learning, synaptic weight should be modulated according to the activity of the CPU4, but in the opposite direction and when the agent is at home. Such bi-directional synaptic modulation for learning and recalibration could be achieved either by a same or different reinforcer neuron (Aso and Rubin, 2016).
- The establishment of a new vector-memory, as well as vector re-calibration, implies long term synaptic change between the hypothesized memory neurons and the CPU4 neurons. Thus, inhibiting long term memory formation in these neurons (e.g., Chen et al., 2012) should prevent the establishment (or re-calibration) of these vector-memories.
- Recall of a vector-memory would simply require the activation of this vector-memory inhibitory neuron, and drive the agent from any location to where the memory has been stored.
- Blocking the activity of such inhibitory neuron should prevent the use of a vector-memory, while driving it should lead the insect to go toward the position in space where the memory has been formed.
- The distributed encoding of vectors in our model provides a simple way to estimate the length of the home vector: by taking the difference in amplitude between the highest and lowest neural activities in the CPU4 integrator layer. Doing so on the resulting vector created by the added inhibitory input of a vector-memory would therefore give a rough estimate of the distance to be covered from the current location to that memory location.

We note that none of these predictions would be trivial to test. However, observing or manipulating the activation of such neural populations in the CX can already be achieved in *Drosophila melanogaster* (Seelig and Jayaraman, 2015; Kim et al., 2017), and local path integration has also been observed in this animal (Kim and Dickinson, 2017). We further hope that modern genetic tools will soon make this endeavor possible in insects such as bees or ants.

## 5. Conclusion

The PI model presented in Stone et al. (2017) was mostly based on identified neurons in the CX, whereas the extensions we have proposed here are speculative. Nevertheless, we have provided a proof of concept that direct return to a salient place, search at this locations, vector recalibration, novel shortcuts and even traplining can emerge given minimal additions to the known CX connectivity. A direction for future work would be to consider how such PI navigation system could be integrated with the use of learnt terrestrial cues, which we know affects how bees and ants behave when homing or returning to a known feeding location (Kohler and Wehner, 2005; Wystrach et al., 2011; Mangan and Webb, 2012; Collett et al., 2013), search at the goal (Schultheiss et al., 2013; Wystrach et al., 2013), take shortcuts from novel locations (Menzel et al., 2005; Collett et al., 2007; Wystrach et al., 2012; Narendra et al., 2013; Cheeseman et al., 2014; Cheung et al., 2014), or form traplines between multiple locations (Ohashi et al., 2006; Lihoreau et al., 2012b). The circuitry of the CX is well suited for such an integration of multiple directional cues (Webb and Wystrach, 2016; Collett and Collett, 2018; Hoinville and Wehner, 2018), and as we show here, for a remarkably rich vector-based navigational repertoire.

## Author Contributions

FL and TS: model implementation; FL, AW, and BW: manuscript writing; FL, ML, AW, and BW: manuscript reviewing; FL, TS, AW, and BW: conceptual ideas.

### Conflict of Interest Statement

The authors declare that the research was conducted in the absence of any commercial or financial relationships that could be construed as a potential conflict of interest.
