# Supplementary material for: The Central Complex as a Potential Substrate for Vector Based Navigation
Source: Front Psychol. 2019 Apr 5;10:690. doi: 10.3389/fpsyg.2019.00690 (PMC6460943; doi:10.3389/fpsyg.2019.00690)
Supplement: Supplementary file 1 [file Data_Sheet_1.PDF]

Supplementary Material: The central complex as a substrate for vector based navigation

1 SUPPLEMENTARY METHODS

1.1 Neurons parameters

Sigmoidal response parameters:

Type	Slope	Bias
TL2	6.8	3.0
CL1	3.0	-0.5
TB1	5.0	0.0
CPU4	5.0	2.5
CPU1	7.5	-1.0
Pontin	5.0	2.5
Motor	1.0	3.0
Vect-mem learning	5.0	2.5

1.2 Neurons connectivity

See Fig. S1 for neurons connectivity details across the CX model.

1.3 Random Walks

Random walks were generated by a filtered noise process, approximating a second order stochastic differential equation (SDE).

$$\begin{aligned}\omega_t &= \lambda\omega_{t-1} + noise \\ \theta_t &= \theta_{t-1} + \omega\end{aligned} \quad \text{(S1)}$$

where ω_t is the angular velocity for the time step t, and where $noise$ is the change in such angular velocity, generated by drawing from a VonMises distribution with the same parameters as described in equation S1.

Here again, the concentration is $\kappa = 100$, smaller values increasing the tortuosity of the outbound route. We used $\lambda = 0.4$, to minimise excessive spiralling motion. Acceleration for outbound routes is generated by drawing evenly spaced ($T/50$) values from a uniform distribution:

$$a \sim U(a_{min}, a_{max}) \quad \text{(S2)}$$

and setting the acceleration between those points using third order spline interpolation, causing the agent to speed up and slow down in a smooth manner, thus imitating natural flight behaviour. This smoothing is

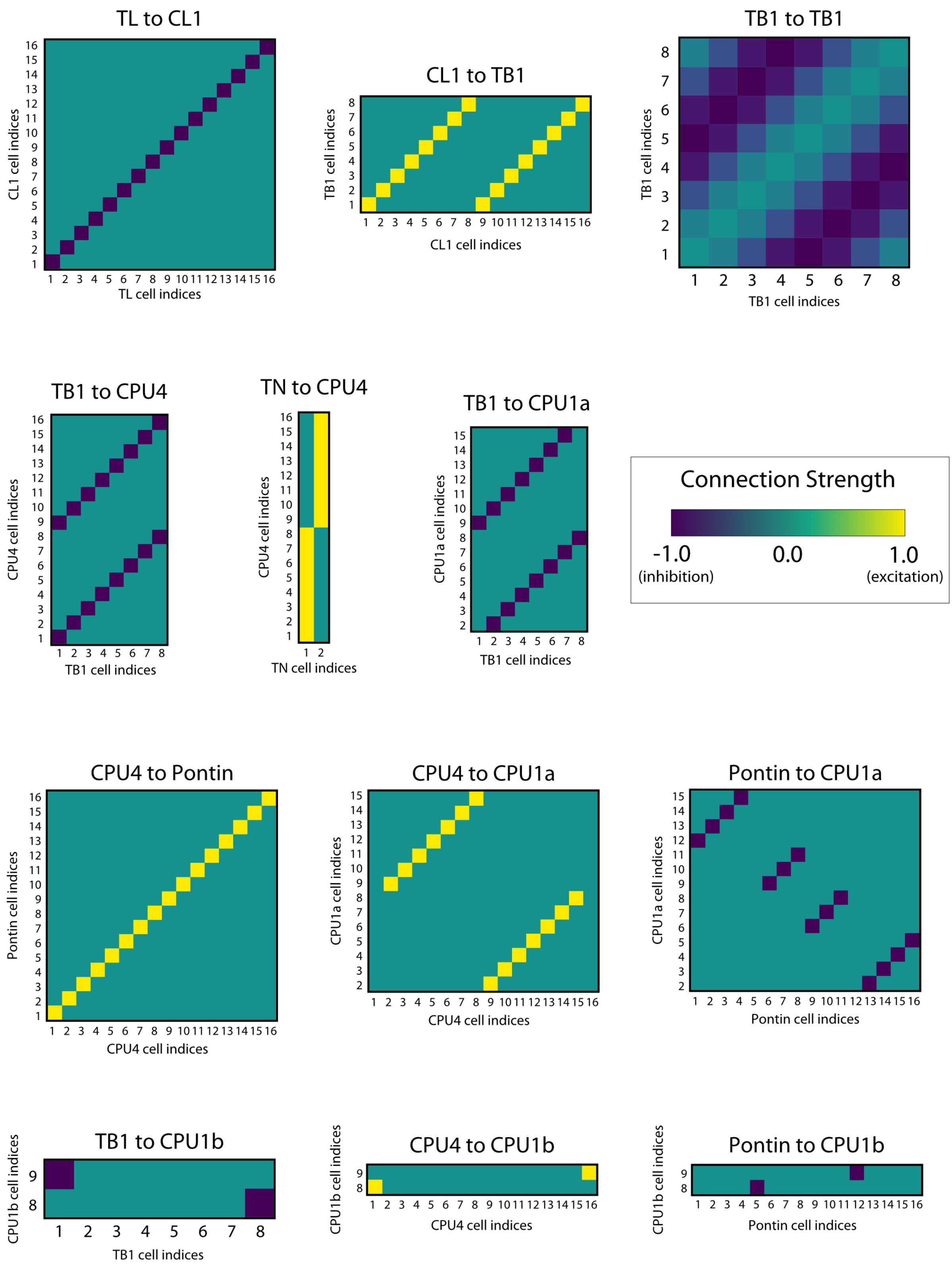

Figure S1. Connectivity matrices between all cell types used in the CX model - Cells synaptic weights are not tuned and are either fully inhibitory ($weight = -1.0$) or fully excitatory ($weight = 1.0$), excepted for the mutually inhibitory interconnections between TB1 neurons (which form a sinusoidal weight matrix crucial for the resulting ring-attractor network). TB1, direction sensitive neurons ; TN, speed-sensitive neurons ; CPU4, integrator neurons ; CPU1, motor output neurons. Also represented for completeness but not crucial for this paper: TL, first polarisation sensitive neurons.

performed in a pre-allocated manner, which means that it does not take into account potential modulation of turning when obstacles are encountered.

Velocity of the agent is determined at each time step by a linear drag model:

$$v = v^{t-1} + \begin{bmatrix} sin(\theta) \\ cos(\theta) \end{bmatrix} \cdot a(1 - F_D) \tag{S3}$$

where $F_D = 0.15$ is the default drag. For regular trials $a_{min} = 0$ and $a_{max} = 0.15$ were tuned to cause v to mostly fall below 0 and 1, allowing the TN cells to capture all speeds without their activity saturating, whereas for inbound paths a is constant with $a = 0.15$. When an object is detected nearby, the drag value is increased to $F_{obj} = 0.30$ to allow efficient modulation of movement.

Note that during random walks, the CX steering layer is getting input only from the heading layer, which results in equal left and right activation and therefore does not affect the movement of the agent.

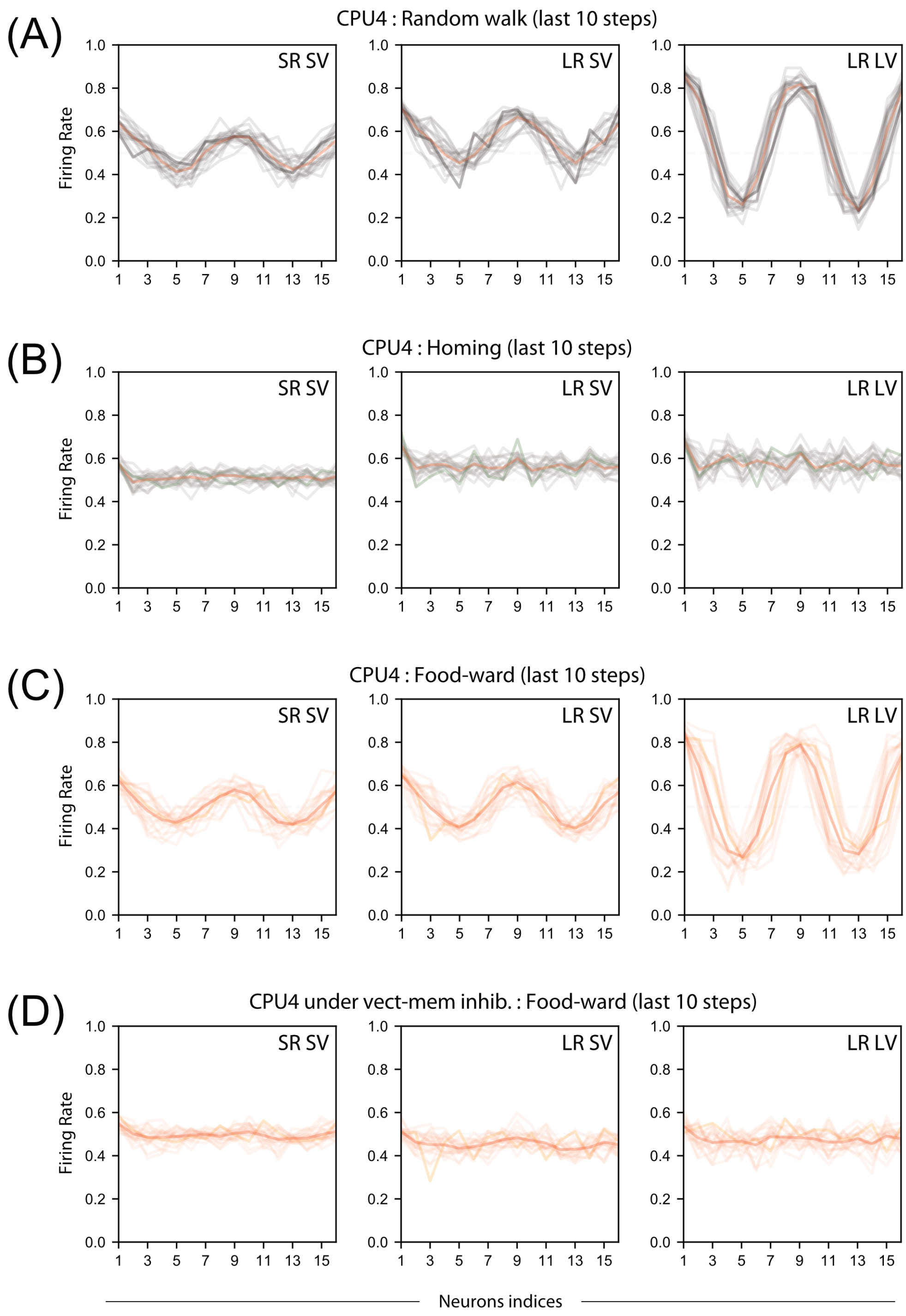

Figure S2. Snapshots of representative cell activities - (A-D) Snapshots of the population activity across the 16 integrator cell outputs, for each of the different tasks. Mean values for each cell across the last 10 steps, for 20 individual trials (phase-aligned as if the direction was the same), and for each of the three groups defined in Fig. S4. Red curves are the mean values over the 20 trials ; grey dotted lines are the baselines (firing rate = 0.5). **(A)** Random walk: For a comparable vector length, the difference in random walk length does not translate a change in amplitude (see SR SV and LR SV). **(B)** Homing: For a comparable vector length, the difference in random walk length induces a positive shift of all the values due to accumulated noise (see SR SV and LR SV). **(C)** Food-ward walk: CPU4 encode the newly walked (memory-driven) path, thus leading to similar curves as in (A), with the exception that noise is eliminated (paths are much shorter than the corresponding random walks). **(D)** Food-ward walk: The inhibited CPU4 output shows a negative shift due to noise present in the LR vector-memories used.

2 SUPPLEMENTARY DATA : PATH ANALYSIS

2.1 Saturation

One major limitation of this model arises because of saturation of the integrator neurons (CPU4) activity. As described in the Methods section as well as in Stone et al. (2017), integrator neurons activity is modelled as noisy sigmoid functions. These neurons charge as the agent moves, and when distance from the nest becomes too important, some of these neurons eventually hit their maximum firing rate and further movement in the same direction can no longer be encoded by saturating neurons.

It seems clear that saturation happens mostly when the distance from the nest goes beyond a certain threshold. Whether this distance is reached in a straight line or after a much longer and sinuous path has little effect (fig. S3, A).

Interestingly, this saturation seems to affect positional error and distance estimation, but not the directional error (fig. S3, C). Direction estimation is unaffected because the general shape of the sinusoid (resulting from the activity of the 8 integrator neurons) and thus its phase, which determine direction, is preserved despite some neurons saturating (fig. S3, B). However, distance estimation is encoded by the amplitude of the sinusoid and thus is directly affected by saturating neurons.

In Homing tasks, the saturation effect can be seen in an incorrect Nest position estimation. In Food-ward tasks, recalling a saturated vector-memory leads to (reversed) saturation in the compound input, producing an estimation error as well.

The major difference between Homing and Food-ward tasks when saturation is present lays in the systematic search displayed at the goal. During homing, the systematic search emerges from the integrator oscillating around its zero-state, whereas during Food-ward trials the systematic search emerges from the compound input of a saturated vector-memory, and a likely a saturated integrator. This translates into an 'all or nothing' type oscillation, making the agent alternate between left and right radical turns, thus completely breaking the search pattern.

Behavioural effects were typically observed when two or more neurons (in the integrator or in the compound input) hit the maximum (1.0) or minimum (0.0) firing rates. This corresponds to a distance of roughly 700 to 800 steps from the Nest. To avoid our results being spoiled by such suturing effects, we established a cut-off and considered in the paper only cases where feeder fell within a radius 700 steps radius from the nest.

2.2 Memory-directed movement: Error analysis

To evaluate our model's performance for the food-ward task, we measured the following:

- The directional error at departure after 50 steps from the nest as the angle between the correct direction and the agent's actual heading direction, a measure often used for bees and ants. We chose 50 steps to ensure that the first corrective turn has been completed. Indeed, as the nest leaving direction is fixed, the agent has to perform a U-turn as soon as it exits the nest when the goal is located on the other side of the nest.
- The positional error in estimating the goal location, as the distance between the goal and the centre of the systematic search displayed, (see 'Systematic search' subsection below).

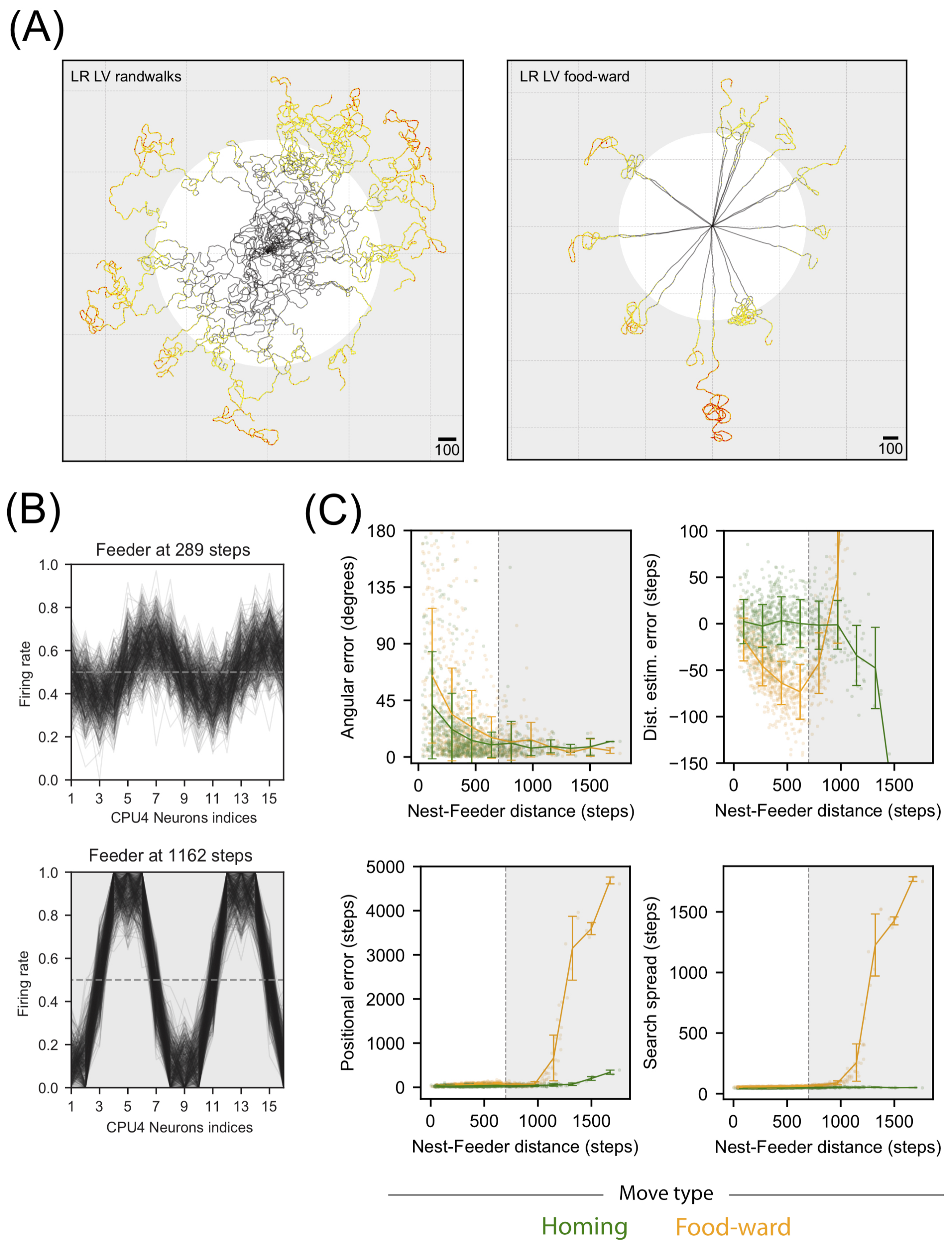

Figure S3. Saturation of the integrator neurons - (A) Left: Traces of individual Long Random walks (LR) that ended at least 1000 steps away from the nest (Long Vector, LV). Nest is the centre point ; Yellow, steps where at least one neuron in the 16 population code saturated (reached the 1.0 or the 0.0 firing rate) ; Orange, at least two neurons saturated ; Red, at least three neurons saturated. Right: Food-ward walks for each of the corresponding Random walks. The one heading south displays characteristic incorrect behaviour due to saturation. This happened typically when at least two neurons in the integrator saturated, hence the cut off at 700 steps we chose for the other experiments (large circle, outer grey area represents the saturation-heavy distances). **(B)** Integrator (CPU4) neurons firing rates for the last 500 steps of food-ward walks where the feeder is either close (top panel), or far away (bottom panel) from the nest. Saturation is clearly visible for the long vector. **(C)** Patterns of error similar to Fig. S4, but without the cut off at 700 steps. Positional and Search spread errors are similar due to the incorrect search pattern as seen in (A), Right (southern trace).

It is important to note that the errors displayed may vary quantitatively depending on the chosen set of parameters, notably the type of random walk used, the type of noise, or the way saturation is implemented (clipping between 0 and 1).

2.2.1 Results

Both the directional error and the positional error were strongly dependent on the absolute distance between the nest and the feeder. For increasing feeder distances, the directional error at departure decreased (fig. S4, A ; linear regression $t = -10.341$, $p < 0.001$, $df = 806$), while the positional error of the goal location increased (fig. S4 B ; linear regression $t = 24.63$, $p < 0.001$, $df = 825$). This was equally true for the homing task (linear regression, directional error: $t = -12.394$, $p < 0.001$, $df = 806$; positional error: $t = 3.672$, $p < 0.001$, $df = 825$). Directional and positional errors were, in average, lower for homing than for returning to the feeder (paired t-test. Difference in directional error: $\mu = -11.81 degrees$ for homing, $t = 5.486$, $p < 0.001$, $df = 805$; difference in positional error: $\mu = -25.06 steps$ for homing, $t = 24.716$, $p < 0.001$, $df = 826$).

The model shows also positional inaccuracies. For instance, the agent tends to systematically underestimate the feeder distance (distance estimation: measured as the difference in length between the estimated and true vector ; t-test against $\mu = 0$: $\mu = -44.97 steps$, $t = -40.279$, $p < 0.001$, $df = 826$). This distance underestimation increased somewhat linearly with feeder distance (fig. S4, C ; $t = -28.586$, $p < 0.001$, $df = 825$). There was no such distance underestimation in the case of homing (fig. S4, C ; t-test against $\mu = 0$: $\mu = 1.29 steps$, $t = 1.580$, $p < 0.114$, $df = 887$).

Also, the agents showed some systematic directional inaccuracies (fig. S5). Random walks strongly biased towards turning on one side (left or right) seems to result in a systematic overestimation of the direction during their subsequent food-ward path (right or left, respectively). There was no such inaccuracies with homing.

Because random walk length and nest-feeder absolute distance are strongly correlated (only long random walks can lead to a distant feeder), we sampled specific cases to disentangle their effects. We selected long random walks that ended up in short absolute nest-feeder distances ($7000 < randwalk < 10,000 steps$, $100 < abs.dist. < 300 steps$, $n = 30$; ”LRSV” for ”Long Random Short Vector”), and compared their errors when returning to the feeder with two alternatives: 1- Short random walks that led to similarly small absolute distances ($100 < randwalk < 2000 steps$, $100 < abs.dist. < 300 steps$, $n = 93$; ”SRSV” for ”Small Random Small Vector”) ; 2- Similarly long random walks that resulted in large absolute distances ($7000 < randwalk < 10,000 steps$, $600 < abs.dist. < 700 steps$, $n = 28$; ”LRLV” for ”Long Random Long Vector”). See fig. S4 boxplots A-B and fig. S2.

Difference between both alternatives thus enables to determine which of random walk length or nest-feeder absolute distance influences most the errors. For outbound paths, directional errors at departure and distance underestimation were overwhelmingly dependent on nest-feeder absolute distance (For directional error, different abs. dist.: $z = 4.457$, $p < 0.001$; different randwalk: $z = 0.133$, $p = 0.894$; Bayesian evidence ratio: 247 in favour of absolute distance. For distance underestimation, different abs. dist.: $z = 4.396$, $p < 0.001$; different randwalk: $z = -0.657$, $p = 0.511$; Bayesian evidence ratio: $12,659$ in favour of random walk length). Positional error depended also on random walk distance, but with more weight given to nest-feeder absolute distance (Positional error: different abs. dist.: $z = -4.069$, $p < 0.001$; different randwalk: $z = 2.600$, $p = 0.009$; Bayesian evidence ratio: 134.04 in favour of absolute distance).

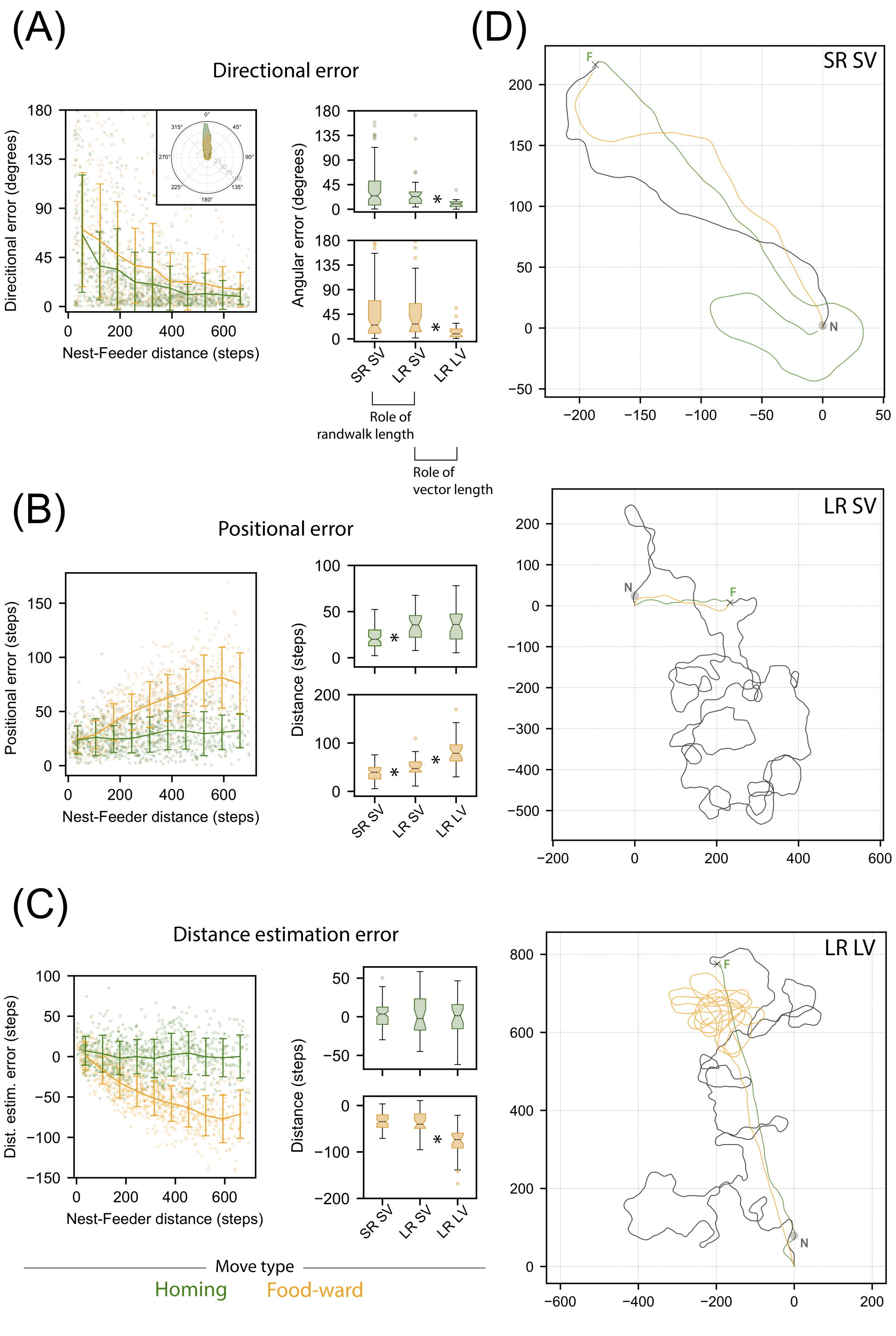

Figure S4. Errors in homing and memory-driven walks - (A-C) Left: Patterns of errors for homing (green) and memory-directed food-ward walk (orange). Solid lines are the mean and standard deviation (10 distance bins). Right: Box-plots corresponding to three defined groups: SR, Small Random walk ; LR, Long Random Walk ; SV, Small Vector : LV, Long Vector. **(A)** Directional error against straight line between Nest and Feeder (polar projections in insets), **(B)** Positional error, **(C)** Distance estimation error (overshoot or undershoot). **(D)** Example traces for each of the three groups. N, Nest ; F, Feeder. Grey trace, random walk. Green trace, homing. Orange trace, memory-driven food-ward walk.

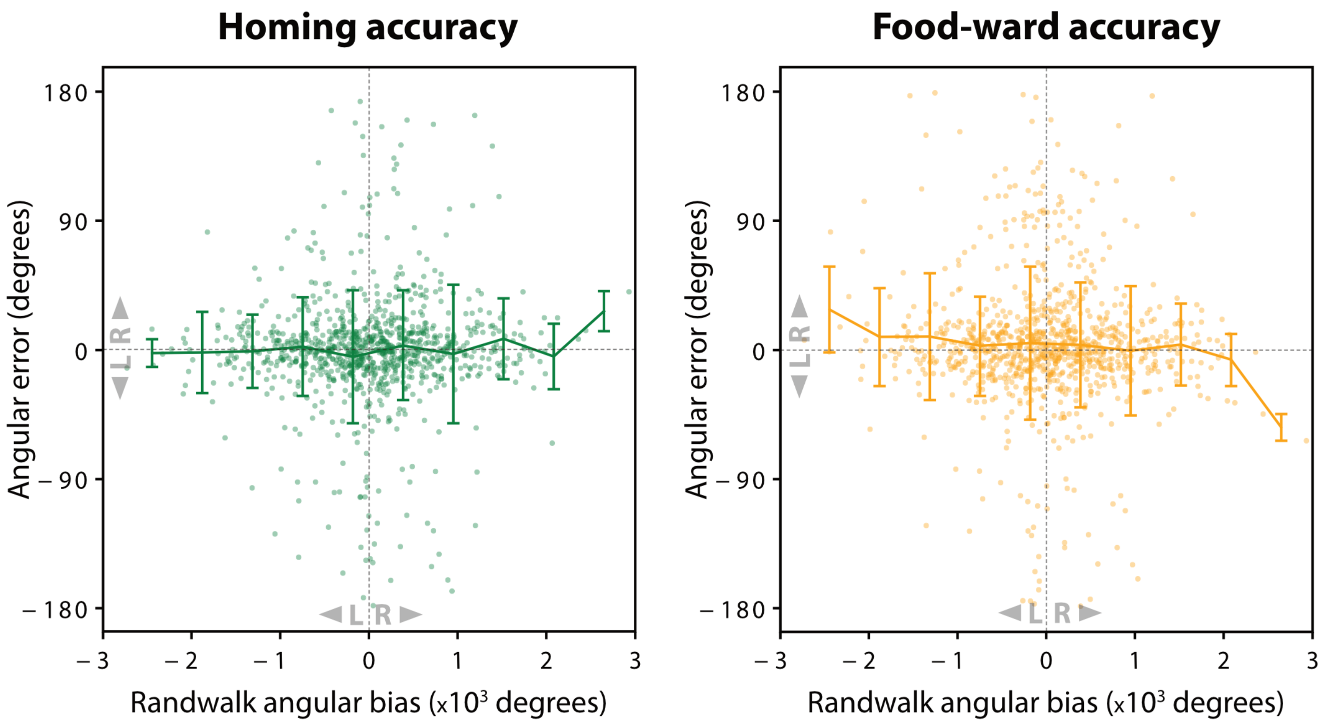

Figure S5. Accuracy of homing and memory-driven walks - Left: Homing. Right: Memory-driven food-ward walk. In both, the angular error of the task is shown against the directional bias of the corresponding random walk. Solid lines are the mean and standard deviation (10 bins).

For homing, directional errors purely depended on absolute nest-feeder distance (different abs. dist.: $z = 3.835$, $p < 0.001$; different randwalk: $z = -0.085$, $p = 0.931$; Bayesian evidence ratio: 1560 in favour of absolute distance), but positional error depended this time only on random walk distance (different abs. dist.: $z = 0.054$, $p = 0.956$; different randwalk: $z = 3.972$, $p < 0.001$; Bayesian evidence ratio: $2,667$ in favour of random walk distance).

2.2.2 Remarks

Directional error at departure was strongly dependent on the feeder distance. Shorter feeder distances yielded higher directional errors, as expected and observed in ants, even though counter-intuitively (Wystrach et al., 2015), and as predicted in a model (Hoinville and Wehner, 2018)."

In our model, this is because short vectors are underpinned by rather small differences in activity across the CPU4 neurons that encode the current state of the PI (or, in an analogous way, in the synaptic weights of the vector-memory neuron). As a consequence, the steering signal is weaker.

We also see that, as previous models predict (Cheung, 2014; Cheung and Vickerstaff, 2010), positional error increases for larger nest-feeder distances, and is dependent on the actual path length. However, there also appears to be some bias (underestimation of distance) in the position (fig. S4, boxplot B), a bias that does not exist in canonical models in absence of memory leakage (Cheung, 2014; Vickerstaff and Cheung, 2010; Cheung and Vickerstaff, 2010); and it is likely that the way noise and saturation are implemented in our neurons, is responsible for it.

The effects of food-nest distance are stronger for food-ward than for homeward routes : while an overall higher error would be expected (and is seen) for food-ward routes, as they always combine the noise of both the vector memory and the current journey, it is not so apparent why the effect of nest-feeder distance should be stronger. It also remains to be seen whether positional uncertainty would decrease with tortuosity as it has been suggested by canonical models (Cheung, 2014).

For homing, the agent did not underestimate goal distance, and the error in estimating the nest position depended on the distance walked as well as feeder distance (fig. S4, C). In ants, it seems that it is only the vector length that influences the positional error (Merkle and Wehner, 2009), however perhaps with very long and convoluted random walks, one could see an effect of walked distance as we observed.

We believe these effects (both underestimation of distance and difference between foodward and homeward journeys) may result from the non-linear activation function used in the model to determine the actual effects of PI memory or vector memory on steering. For longer distances, the baseline for memory activity tends to drift towards 1, as it is not fully compensated by the constant leak. The non-linearity represented by the sigmoid function produces greater distortion near 1. Moreover, a vector memory stored for a longer distance will contribute this distortion to every step of a foodward path, whereas for homeward PI, the memory activity will tend to decrease, reducing the effect of sigmoidal distortion as home is approached.

In the extreme case for longer distances, the upper bound of 1 imposed on the neural activation produces even more pronounced effects, as saturation distorts the representation of the vector by 'clipping' the top of the sine wave. Interestingly, this should affect estimation of distance more than estimation of direction, although in the limit, both are catastrophically affected.

2.3 Search spread analysis

If the agent is left to run once arrived at the goal (i.e. goal removed), it spontaneously displays a search pattern around the expected goal location (fig. S6 and fig. S4, trace C). In order to look at this systematic search, we ran $N = 1,000$ trials using the 1,000 vector-memories/goal coordinates previously generated. We discarded time steps from $t = 0$ to $t = (2 \times D_o)$, where D_o is the straight-line distance between the nest and the goal. The systematic search's spread and barycentre coordinates were analysed.

2.3.1 Results

The spread (measured as the average distance away from the search's barycentre) was strongly dependent on the feeder distance. For increasing feeder distances, the spread of the search for the feeder increased (fig. S6, A ; linear regression: $t = 19.81$, $p < 0.001$, $df = 825$). This was equally true, but to a lesser extent, for the homing task (fig. S6, A ; linear regression: $t = 11.47$, $p < 0.001$, $df = 825$). Searches at the feeder were, in most cases, looser than the nest searches (fig. S6, B-C ; paired t-test, difference in spread: $\mu = -8.61$ steps for homing, $t = 66.891$, $p < 0.001$, $df = 825$). This difference in spread showed a significant interaction with goal distance ($t = 8.30$, $p < 0.001$, $df = 825$) because the increased in spread with feeder distance is quicker for feeder searches (fig. S6, A).

We sampled here again the same specific cases to disentangle the effects of random walk length and nest-feeder absolute distance on search spread. We selected long random walks that ended up in short absolute nest-feeder distances ($7000 < randwalk < 10,000 steps$, $100 < abs.dist. < 300 steps$, $n = 30$; "LRSV" for "Long Random Short Vector"), and compared their search spread when returning to the feeder with two alternatives: 1- Short random walks that led to similarly small absolute distances ($100 < randwalk < 2000 steps$, $100 < abs.dist. < 300 steps$, $n = 93$; "SRSV" for "Small Random Small Vector") ; 2-

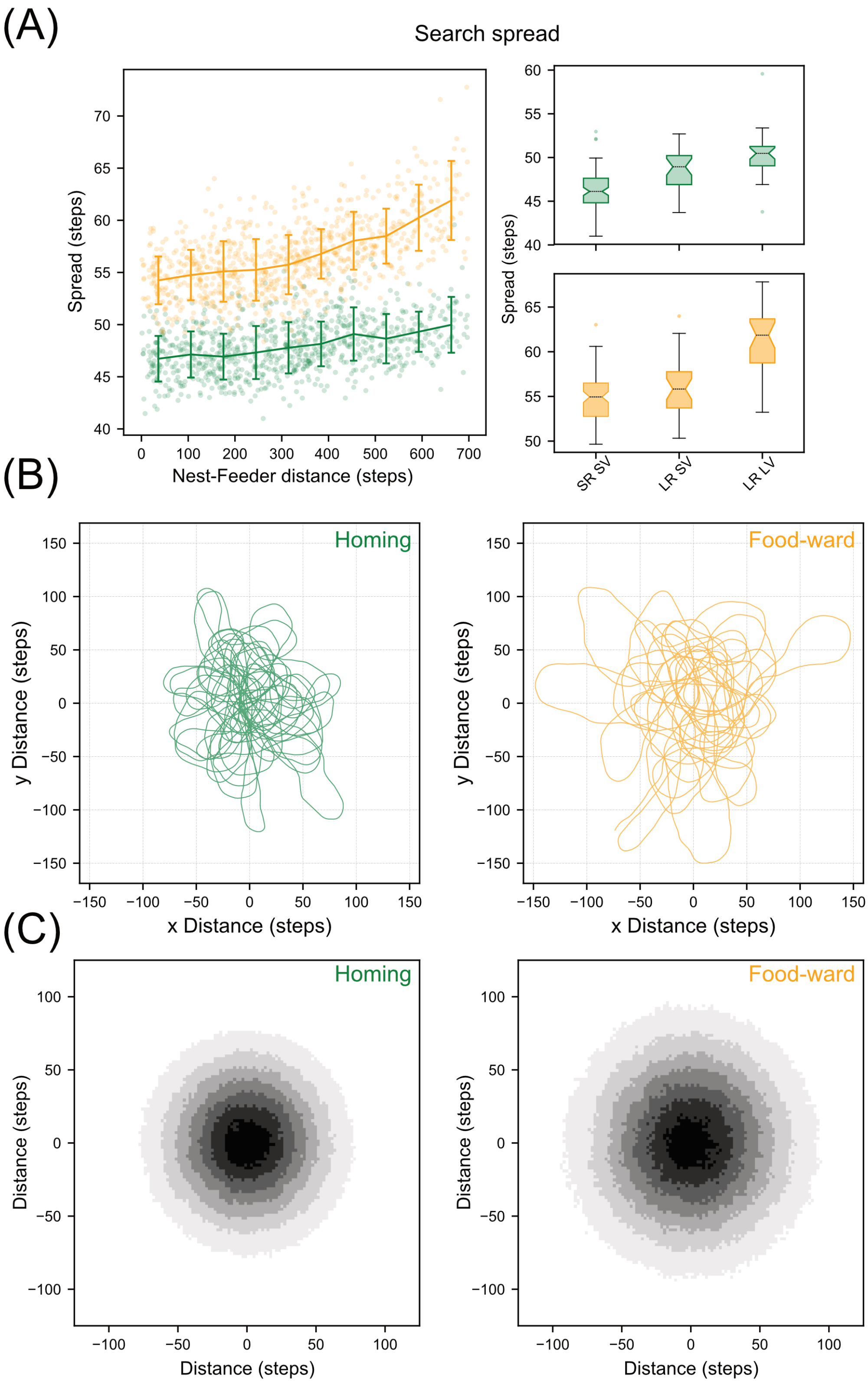

Figure S6. Systematic search - (A) Left: Scatter plot of the spread of the systematic search around the goal for n = 1000 individual trials. Green, homing ; Orange, memory-driven food-ward walk. Solid lines are the mean and standard deviation (10 distance bins). Right: Box-plots of the systematic search spread for the same three groups as in Fig. S4. **(B)** Example traces of two individual systematic searches (re-centred to the origin for clarity), one around the Nest (Left, green), and one around the Feeder (Right, orange). **(C)** 2D Histogram of the spread of the systematic search for the n = 1000 trials, all re-centred to the origin.

Similarly long random walks that resulted in large absolute distances ($7000 < randwalk < 10,000steps$, $600 < abs.dist. < 700steps$, $n = 28$; "LRLV" for "Long Random Long Vector"). Difference between both alternatives thus enables to determine which of random walk length or nest-feeder absolute distance influences most the errors (fig. S6, A box-plots).

While search spread at the feeder was mostly dependent on nest-feeder absolute distance (different abs. dist.: $z = -5.142$, $p < 0.001$; different randwalk: $z = 1.705$, $p < 0.088$; evidence ratio: $129,375$ in favour of nest-feeder absolute distance), search spread at the nest depended also on random walk length (different abs. dist.: $z = -2.575$, $p = 0.010$; different randwalk: $z = 4.620$, $p < 0.001$; evidence ratio: $1,568$ in favour of random walk length).

2.3.2 Remarks

The systematic search displayed by the agent around the expected goal location is reminiscent to what is observed with ants (Wehner and Srinivasan, 1981; Schultheiss and Cheng, 2011). Conditions with high positional uncertainty, such as searching at a distant feeder, produce broader search patterns ; while conditions with low positional uncertainty, such as searching for the nest after a short walk produce tighter searches (fig. S6).

The mechanistic correlates underlying these errors seem quite clear. In our model, absolute distance from the nest is encoded as large amplitude difference in activity across the cell population, whereas longer distances walked result in an increase in overall activity (fig. S2). Both effects seem to have a negative impact on position estimation. Because large amplitude in activity makes the neurons less sensitive, assuming their activity follow a sigmoid function (Hertz et al., 1991), distance encoding is thus slightly 'underestimated' when far from the nest (or after a very long walk). In addition, the effect on search spread suggests that high amplitude in neural activity also impacts guidance directly. For instance, searching at a distant feeder is achieved in the context of large amplitude in the neural activity of both the CPU4 and the inhibitory vector-memory neuron, resulting in large positional error and large search spread.

Note that for very large distances, the PI representation in our model saturates, disrupting the system and causing a sudden exponential increase of errors (S3). Insects may have a way to prevent this kind of saturation, perhaps by simply avoiding going too far (Muser et al., 2005; Wehner et al., 2004).

2.4 Shortcutting: Error analysis

We investigated how the errors of the shortcut related to the spatial relationship between the nest and the feeders. To tell apart the effects of inter-feeders and nest-feeders distances, we run a multivariate model taking into account the distance between Feeder1-Feeder2 ; Nest-Feeder1 ; Nest-Feeder2 and their interactions. In our sample, these variables were relatively independent from each other. We assessed the effect of the distance between the two feeders on success by generating 1000 goals couples with different inter-feeder distances ranging from 100 to 1400 steps. We then only considered trials where both feeders were within the radius of 700 steps of the nests.

2.4.1 Results

Failure in reaching Feeder 2 happened typically when the second feeder was particularly far from the nest (mean $\pm$ std nest-feeder2 distance: 564.35 ± 108.82 steps, top 87.04%), whereas inter-feeder distance was rather average (mean $\pm$ std inter-feeder distance: 505.04 ± 355.51 steps, top 57.51%).

The directional error at departure from Feeder 1 appeared only dependent on the Feeder 1-Feeder 2 distance (fig. S7 A, linear regression: F1-F2: $t = -3.015$, $p = 0.003$; N-F1: $t = -0.626$, $p = 0.532$;

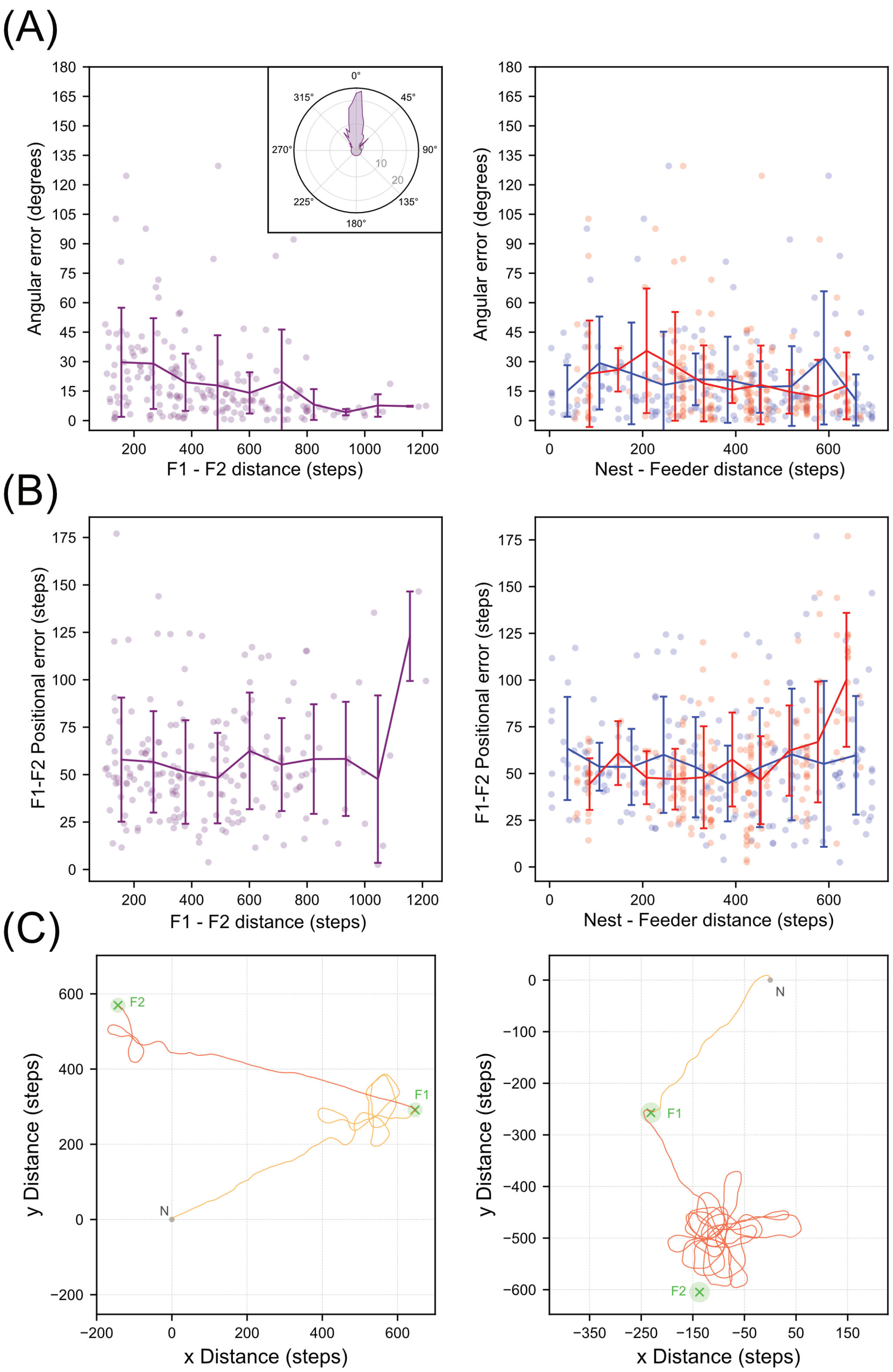

Figure S7. Errors in the shortcutting task - (A) Left: Scatter plots of the directional error against the straight-line distance between Feeder 1 and Feeder 2 (purple data) ; Right: Directional error against the Nest-Feeder 1 (blue data) and Nest-Feeder 2 (red data) straight-line distances. Solid lines are the mean and standard deviation (10 distance bins). Left inset: Circular visualisation of the angular error. **(B)** Positional error in locating the Feeders during the shortcutting task. Colours as in A. **(C)** Example traces of shortcuts. Left: successful shortcut (Feeder 1 and Feeder 2 are found). Right: Failed shortcut (Feeder 2 not found due to directional and positional error).

N-F2: $t = -0.251, p = 0.801$; $df = 185$). The shorter the inter-feeder distance, the higher the directional error. The error in estimating the goal (Feeder 2) position, however, was dependent on the Nest-Feeder2 distance (fig. S7 B, linear regression: F1-F2: $t = -1.269, p = 0.206$; N-F1: $t = 0.030, p = 0.976$; N-F2: $t = 4.760, p < 0.001$; $df = 185$). Positional error increased as Feeder 2 was further away from the nest.

The pattern of errors observed during these shortcuts can be explained by the same mechanisms discussed above for food-ward or homing journeys. Directional errors (when leaving the first feeder) increases as feeder1-feeder2 distance shortens (fig. S7, A), because small shortcut distances result in small differences in activity in the compound state, yielding a weak steering signal. Errors in estimating the position of the feeder2 increase as the nest-feeder2 distance increases (fig. S7, B), because of the deleterious effect of large neural activity amplitudes that arise when far away from the nest (fig. S7).

2.5 Multi-location routes

It is worth noting that we generated the memories and tested these memories at two different scales: a smaller scale was used for vector memory generation, which allowed for quicker discovery and decreased simulation time. We then tested these memories at a bigger scale: all environment dimensions, catchment areas, and vector memory profiles were multiplied by the same constant. This allows for a better visibility of the agent's path, since when all dimensions are smaller, the agent's actual path tends to be 'diluted' by the noise, and memories are consumed immediately, leaving only the random search pattern to appear, which causes the feeder discovery to be more random-driven than memory-driven. The constant with which we scaled the experiment was chosen for each feeders array so that the furthest feeder was always around 600 steps in distance from the nest, to stay in the optimal distance range where neither saturation nor dilution effect can occur. Feeders and Nest catchment areas were not concerned by the scaling and were fixed to a radius of 20 steps after as it is throughout all our experiments.

2.5.1 Sequences

Positive array (5 feeders):

Sequences	Successful trials	Remarks
5, 4, 3, 2, 1	77.71%	Nearest neighbour and Optimal route
1, 2, 3, 4, 5	15.07%	Nearest neighbour and Optimal route
5, 3, 4, 2, 1	1.91%	
1, 5, 4, 3, 2	1.49%	Nearest neighbour
4, 3, 2, 1, 5	1.27%	
5, 1, 2, 3, 4	0.64%	Nearest neighbour
Others	1.91%	$< 1\%$ each

Negative array (6 feeders):

Sequences	Successful trials	Remarks
1, 2, 4, 3, 5, 6	47.23%	Second optimal route
1, 2, 4, 5, 6, 3	41.28%	Nearest neighbour
2, 1, 4, 5, 6, 3	3.62%	
2, 1, 4, 3, 5, 6	3.40%	
1, 2, 3, 4, 5, 6	2.77%	Optimal route
Others	1.70%	$< 1\%$ each

Negative array (10 feeders):

Sequences	Successful trials	Remarks
1, 3, 4, 5, 6, 7, 8, 9, 10, 2	2.31%	
1, 2, 4, 5, 6, 9, 8, 10, 7, 2	1.47%	
1, 3, 4, 5, 6, 7, 8, 10, 9, 2	1.05%	
1, 2, 5, 4, 7, 8, 9, 10, 6, 3	1.05%	Optimal route
1, 3, 4, 7, 8, 10, 9, 6, 5, 2	0.63%	Optimal route
2, 5, 6, 9, 10, 8, 7, 4, 3, 1	0.21%	Optimal route
3, 4, 7, 8, 10, 9, 6, 5, 2, 1	0.21%	Optimal route
Others	93.07%	$< 1\%$ each

2.5.2 Remarks

Our agent started to display an optimal route around the 22nd trial in average, as do bees in this array (around 18 trials) (Lihoreau et al., 2012). It took however more trials (20 trials) for our agent to find all the flowers than it took for the bees (9 trials), but this could be tuned in our model by improving the random search or letting our agent search for longer. There was usually only 1 or 2 trials between the moment where all flowers have been discovered and the establishment of an optimal route. In bees, the delay between the discovery of all the flowers and the establishment of an efficient multi-feeder route is longer (around 10 trials), which might be expected if one assumes that bees also learn the visual route segments between flowers. It is worth noting that in this flower array, the agents tended to alternate between the two optimal routes (clockwise and anti-clockwise). This is because, in each bout, as the vector-memories were updated, the one of the last visited flower is slightly higher in activity overall (because at the end of the route), which makes it more likely to be used first in the next bout due to the negative shift induced by the added noise (see fig. S2, B and D). But one would expect that this effect would be overridden simply by considering the learning of terrestrial cues along the route. However, and perhaps more surprisingly, swapping between clockwise and anti-clockwise route has also been observed in bumblebees (see Lihoreau et al. (2012, Table S1)). An additional concept not yet included in our model would be to alter the probability of either storing or recovering the vector-memory of a feeder depending on the density or quality of food discovered (Bolek et al., 2012; Wolf et al., 2012; Lihoreau et al., 2011)

REFERENCES

Bolek, S., Wittlinger, M., and Wolf, H. (2012). Establishing food site vectors in desert ants. *Journal of Experimental Biology* 215, 653–656

Cheung, A. (2014). Animal path integration: a model of positional uncertainty along tortuous paths. *Journal of theoretical biology* 341, 17–33

Cheung, A. and Vickerstaff, R. (2010). Finding the way with a noisy brain. *PLoS computational biology* 6, e1000992

Hertz, J., Krogh, A., and Palmer, R. G. (1991). *Introduction to the theory of neural computation.* (Addison-Wesley/Addison Wesley Longman)

Hoinville, T. and Wehner, R. (2018). Optimal multiguidance integration in insect navigation. *Proceedings of the National Academy of Sciences* 115, 2824–2829

Lihoreau, M., Chittka, L., and Raine, N. E. (2011). Trade-off between travel distance and prioritization of high-reward sites in traplining bumblebees. *Functional Ecology* 25, 1284–1292

Lihoreau, M., Raine, N. E., Reynolds, A. M., Stelzer, R. J., Lim, K. S., Smith, A. D., et al. (2012). Radar tracking and motion-sensitive cameras on flowers reveal the development of pollinator multi-destination routes over large spatial scales. *PLoS biology* 10, e1001392

Merkle, T. and Wehner, R. (2009). How flexible is the systematic search behaviour of desert ants? *Animal Behaviour* 77, 1051–1056

Muser, B., Sommer, S., Wolf, H., and Wehner, R. (2005). Foraging ecology of the thermophilic australian desert ant, melophorus bagoti. *Australian Journal of Zoology* 53, 301–311

Schultheiss, P. and Cheng, K. (2011). Finding the nest: inbound searching behaviour in the australian desert ant, melophorus bagoti. *Animal Behaviour* 81, 1031–1038

Stone, T., Webb, B., Adden, A., Weddig, N. B., Honkanen, A., Templin, R., et al. (2017). An anatomically constrained model for path integration in the bee brain. *Current Biology* 27, 3069–3085

Vickerstaff, R. J. and Cheung, A. (2010). Which coordinate system for modelling path integration? *Journal of Theoretical Biology* 263, 242–261

Wehner, R., Meier, C., and Zollikofer, C. (2004). The ontogeny of foragwehaviour in desert ants, cataglyphis bicolor. *Ecological Entomology* 29, 240–250

Wehner, R. and Srinivasan, M. V. (1981). Searching behaviour of desert ants, genuscataglyphis (formicidae, hymenoptera). *Journal of comparative physiology* 142, 315–338

Wolf, H., Wittlinger, M., and Bolek, S. (2012). Re-visiting of plentiful food sources and food search strategies in desert ants. *Frontiers in neuroscience* 6, 102

Wystrach, A., Mangan, M., and Webb, B. (2015). Optimal cue integration in ants. *Proc. R. Soc. B* 282, 20151484
